# Association of reproductive factors with dementia: A systematic review and dose-response meta-analyses of observational studies

**DOI:** 10.1016/j.eclinm.2021.101236

**Published:** 2021-12-14

**Authors:** Chunying Fu, Wenting Hao, Nipun Shrestha, Salim S. Virani, Shiva Raj Mishra, Dongshan Zhu

**Affiliations:** a Centre for Health Management and Policy Research, School of Public Health, Cheeloo College of Medicine, Shandong University, Jinan, 250012, China; b NHC Key Lab of Health Economics and Policy Research (Shandong University), Jinan, 250012, China; c Department of Primary care and mental health, University of Liverpool; d Michael E. DeBakey VA Medical Center and Baylor College of Medicine, Houston, TX, United States of America; e Academy for Data Sciences and Global Health, Kathmandu, Nepal; f Salim Yusuf Emerging Leaders Program, World Heart Federation, Geneva, Switzerland

**Keywords:** Menarche, menopause, Reproductive period, Estrogen level, Dementia, Cognitive impairment

## Abstract

**Background:**

Associations between endogenous estrogen exposure indicators and risk of subtypes of dementia have been unclear.

**Methods:**

Databases (PubMed, EMBASE and Web of Science) were searched electronically on 1st July and updated regularly until 12nd November 2021. Observational studies of English language were selected if reported an effect estimate [e.g., odds ratio (OR), rate ratio (RR) or hazard ratio (HR)] and 95% CI for the association between any exposure (age of menarche, age at menopause, reproductive period, estradiol level) and any endpoint variable [all-cause dementia, Alzheimer's disease (AD), vascular dementia (VD), cognitive impairment (CI)]. Random-effects models and dose-response meta-analyses were used to calculate estimates and to show the linear/nonlinear relationship. PROSPERO CRD42021274827.

**Findings:**

We included 22 studies (475 9764 women) in this analysis. We found no clear relationship between late menarche (≥14 vs <14 years) and dementia, CI in categorical meta-analysis compared to a J-shape relationship in dose-response meta-analyses. Later menopause (≥45 vs <45 years) was consistently associated with a lower risk of all-cause dementia (pooled RR: 0.87, 95%CI: 0.78–0.97, I^2^=56.0%), AD (0.67, 0.44–0.99, I^2^=78.3%), VD (0.87, 0.80–0.94) and CI (0.82, 0.71–0.94, I^2^=19.3%) in categorical meta-analysis, showing similar results in dose-response meta-analyses. An inverse relationship between longer reproductive duration (≥35 vs <35 years) and dementia was observed in dose-response meta-analysis. In addition, estradiol levels after menopause were inversely correlated with the risk of AD and CI.

**Interpretation:**

In this study, later menopause and longer reproductive period were associated with a lower risk of dementia, while the relationship for menarchal age was J-shaped. There was an inverse relationship between higher postmenopausal estrogen levels and risk of AD and CI. Longitudinal study are needed to further explore the association between life-time estrogen exposure and risk of subtypes of dementia.

**Funding:**

Start-up Foundation for Scientific Research in Shandong University.

**Research in context**

**Evidence before this study**

Previous studies have shown inconsistent findings on the associations between age at menopause, length of reproductive period and risk of dementia.

**Added value of this study**

Compared with previous studies, we included both pre- and postmenopausal estrogen exposures in the present review, providing a life-course perspective into understanding the relationship between endogenous estrogen exposure and subtypes of dementia. Methodologically, besides using categorized meta-analyses, we also used dose-response meta-analyses to show the linear/non-linear relationship between the exposure and outcome across a continuous exposure spectrum.

**Implications of all the available evidence**

Later menopause and longer reproductive period were associated with a lower risk of dementia, while the relationship for menarchal age was J-shaped. Higher concentration of endogenous estradiol after menopause was linked to lower risk of AD and CI. Longitudinal, repeat measure designs are needed to examine the association between life-time estrogen exposure and risk of subtypes of dementia using direct measure of serum level of endogenous estrogen before and after menopause.

Alt-text: Unlabelled box

## Introduction

Sex differences have been shown in the epidemiology of dementia.^1^ Compared to men of the same age, women aged 60 to 69 years showed 1.9 times higher prevalence (108 cases versus 56 cases per 10,000 persons) of Alzheimer's disease (AD) than men, while the prevalence of vascular dementia (VD) was 1.8 times higher (56 cases versus 32 cases per 10,000 persons) in men than in women.^2^ Also, women at all ages after age 60 showed higher prevalence of mild cognitive impairment (MCI) than men.^3^ These differences were not fully explained by women's higher longevity and different burden of traditional risk factors in both sex (e.g., low literacy, physical inactivity).^4^^,^^5^ Evidence has suggested that conditions related to pregnancy, breastfeeding, parity, menopause and estrogen level were linked to risk of AD, and they might be female-specific risk-enhancing factors.^6^, ^7^, ^8^ These reproductive factors throughout women's lifespan might contribute to the elevated risk of dementia in women, and serve as a pivotal times to assess their risk of dementia.

Age at menopause, age at menarche and reproductive period are all indicators of endogenous estrogen exposure before menopause. Previous studies have shown inconsistent findings on the associations between these indicators and risk of dementia. Late age at menarche had been linked to poor cognitive function or elevated risk of dementia,^9^^,^^10^ while one study found no association between them.^11^ Late age at menopause has been associated with both lower^6^^,^^7^ and higher^12^^,^^13^ risk of dementia whilst some studies have found no association.^11^^,^^14^ Reproductive period can be described as time from age at menarche to age at menopause.^12^^,^^15^ The relationship between length of reproductive period and dementia or cognitive impairment (CI) is also inconclusive. Some studies have shown longer reproductive period was associated with reduced risk of dementia or CI^10^^,^^16^ while other studies have shown increased risk.^12^^,^^13^^,^^17^ In addition, from a life course perspective, estrogen level after menopause might be also linked to cognitive decline. There were few studies on the relationship between postmenopausal level of endogenous estrogen and dementia. Evidence on the association of concentration of estrogen after menopause and risk of dementia remains unclear, and most of findings were from cross-sectional studies.^18^, ^19^, ^20^, ^21^, ^22^

The aim of this study was to synthesize and quantify the association of reproductive factors: age at menarche, age at menopause, reproductive period, postmenopausal level of estrogen with risk of all-cause dementia, Alzheimer's disease (AD), vascular dementia (VD), and CI.

## Methods

We followed the Preferred Reporting Items for Systematic Reviews and Meta-analyses (PRISMA) and Meta-analysis of Observational Studies in Epidemiology (MOOSE) reporting guidelines. This study was registered with PROSPERO, CRD42021274827.

### Search strategy and data extraction

Three online databases, PubMed, EMBASE and Web of Science were searched using a combination of search terms as following up to 1st July 2021.

The search strategy included combined terms on: (1) terms related to menarche (“age at menarche”, menarch*, pubert*, “sexual maturation”, precocious, Menarche [Mesh], puberty [Mesh], sexual maturation [Mesh]); (2) terms related to menopause (“age at menopause”, menopaus*, climacteric, perimenopaus*, postmenopaus*, “onset menopause”, “age at natural menopause”, “final menstrual period”, “final menstruation”, Menopause [Mesh], Climacteric [Mesh], Perimenopause [Mesh], Postmenopause [Mesh]); (3) terms related to reproductive period (“reproductive timing”, “reproductive time”, “reproductive duration”, “reproductive year*”, “reproductive history”, “reproductive span*”, “reproductive life span*”, “reproductive period”); (4) terms related to estrogen exposure (“Estrogen”, “Oestrogen”, “estradiol”); (5) terms related to cognitive impairment (Dementia, “Cognitive capabilit*”, “Alzheimer's disease”, “Cognitive impairment”, “Cognition function”, “cognitive function”, Alzheimer). The detailed search strategy for each database was listed in eMethods in the Supplement.

First, we imported all searched literature into Endnote and excluded duplicates. The search was limited to studies on human beings and were published in English. Then two investigators screened the titles and abstracts independently and selected the final list of studies in consensus (WTH and CYF). Finally, we extracted the first author's name, country, study design, exposure variables, outcome variables, covariates adjusted, estimates (e.g., adjusted odds ratio (OR), relative risk (RR) or hazard ratio (HR)) and their 95% CIs. The search was updated on 12nd November 2021.

### Study selection

We included studies if they met the following inclusion criteria. (1) Observational studies. (2) Exposure variable was at least one of the following: age at menarche, age at menopause, reproductive duration, or concentration of endogenous estrogen after menopause. (3) The endpoint of interest was all-cause dementia, AD, VD, or CI. (4) The study reported an effect estimate (e.g., OR, RR or HR) and the corresponding 95% confidence interval (95% CI) for the association between exposure and endpoint variable.

### Exposures of interest

We defined four exposure variables of interest: (1) Age at menarche was the occurrence of a first menstrual period in female adolescence^23^; (2) Age at menopause was defined retrospectively as the cessation of spontaneous menses for 12 months^24^; (3) Reproductive period was the difference between age of menopause and age of menarche^25^; (4) Concentration (pmol/L) of serum estradiol level after menopause was used to indicate postmenopausal estrogen exposure.

### Outcomes

The outcomes of interest were the following: all-cause dementia, AD, VD and CI. The presence of dementia, AD and VD was determined by self-reported dementia or AD or VD diagnosed by a doctor, ascertained by medical records. The presence of CI (including MCI) or not was assessed by using scales of Mini-mental State Examination (MMSE) or Montreal Cognitive Assessment (MoCA). MMSE<24 or education-specific cut-off points of MMSE (score 17/18 for subjects with no education, 20/21 for subjects with primary school education, and 24/25 for those with secondary school or higher education), and MoCA<26 were used to define CI. MMSE<27 was used to define MCI.

### Risk of bias assessment

The Newcastle-Ottawa Scale (NOS) was used to assess risk of bias for systematic review of observational studies.^26^ The NOS is an eight-item instrument designed to rate methodological aspects of case-control and cohort studies. A study was given a maximum of one score for items under selection and outcome domain, and two scores under comparability domain. The overall score ranges from 0 to 9 for each study. A score of 0–5, 5–6 and 7–9 was rated as low, moderate and high-quality studies, respectively.^27^ The Agency for Healthcare Research and Quality (AHRQ) scale was used to assess the quality of cross-sectional studies.^28^ There were 11 items in total, and each item has three options of yes, no, and not clear. The answer “Yes” will score 1 point, and the answer “No or Not Clear” will score 0 point. The overall score ranges from 0 to 11. A score of 0–3, 4–7 and 8–11 was rated as low, moderate, and high-quality studies respectively.^29^

### Statistical analysis

Given the lower than 10% incidence of dementia or MCI (e.g., dementia: 9.87–17.18/1000 person-years,^30^^,^^31^ AD: 2.2% (95% CI: 1.6–2.8),^32^ MCI: 22.6 (95% CI: 19.6–25.9) and 8.67 (95% CI: 7.0–10.7) per 1000 person-years for less severe and severe cognitive impairment respectively^33^), we approximated HR and OR as RR when pooling the estimates across the studies.^34^

Two types of meta-analysis were performed. We first performed traditional meta-analyses (of categorical exposure variable) to yield a pooled estimate of the association of menarchal age, menopausal age, reproduction duration with each outcome (all-cause dementia, AD, VD, and CI), respectively. Forest plots were used to show results. As categories of exposure variables differed in individual studies and lacked a uniform reference, we recombined the original categories into simplified categories to pool the effect estimates. For example, age at menopause categories: <40, 40–44, 45–49, 50–54, ≥55 years were combined as <45 and ≥45 years. Categorical meta-analysis was not conducted for estradiol level due to large variation between studies. Limited studies precluded further analysis of the association between reproductive factors and VD.

Second, we conducted a dose-response meta-analysis for each exposure-outcome relationship. Step one, we performed a non-linear dose-response meta-analysis for each exposure-outcome relationship, then based on the *χ^2^* and *p*-value calculated in step one, we determined whether a linear (*P*>0.05) or non-linear (*P*<0.05) dose-response meta-analysis should be adopted. The *Q* and *I^2^* statistics were used to evaluate the heterogeneity among studies. Higgins et al. (2003) suggested that heterogeneity could be quantified as low, moderate, and high to I^2^ values of 25%, 50%, and 75%, respectively.^35^ Subgroup analysis was performed to investigate sources of heterogeneity by race (white and non-white) and study design (case-control or cohort). Random-effect models were used to pool the RR.

Finally, we performed subgroup analyses based on study design (case-control or cohort) and race (white and non-white). Additionally, to address potential bias and verify our results, we performed various sensitivity analyses by (1) excluding low-quality studies, (2) using a leave-one-out method, (3) Mantel-Haenszel weighting. To investigate the risk of publication bias, we applied the Egger test and visually inspected the funnel plots.

All analyses were carried out using Review Manager, version 5.4 (Nordic Cochrane center), complemented by STATA statistical software, version 15.0 (StataCorp). Generalized least squares for trend estimation (GLST) function was used to conduct dose-response meta-analysis. All statistical tests were based on the two-sided 5% level of significance.

### Role of the funding source

The funders had no role in study design, data collection, data analysis, data interpretation, or writing of the report. The corresponding author had full access to all the data in the study and final responsibility for the decision to submit for publication.

## Results

### Study selection

**Figure 1:**
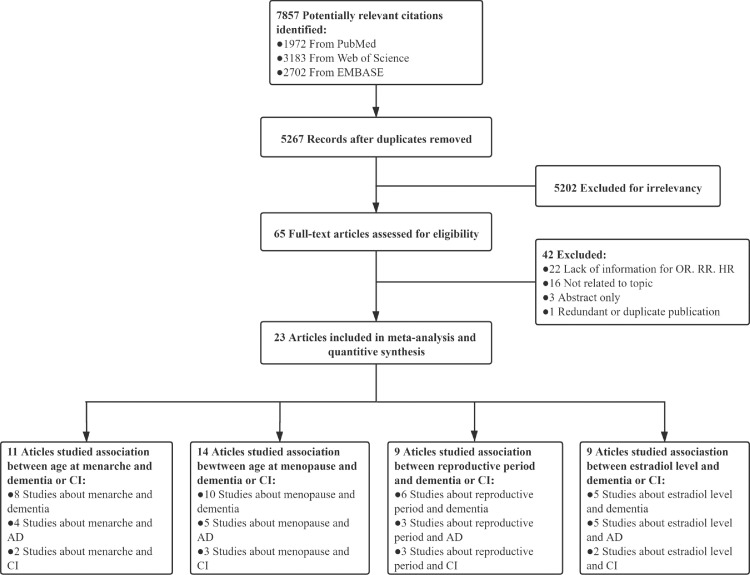
flowchart of included studies.

We identified 7 857 studies from various databases initially. Among them, 7523 were excluded for duplication or for not meeting our inclusion criteria. Thus, 64 studies were left for full paper assessment. Of them, 19 (43.2%) studies lacked information of effect sizes cannot be calculated based on the data provided, 16 (36.3%) were not related to the topic, three (6.8%) had abstract only, three (6.8%) included women using exogenous estrogen therapy, one (2.2%) was a duplicate publication.^36^ Finally, 22 articles^6^^,^^11^, ^12^, ^13^^,^^16^, ^17^, ^18^^,^^21^^,^^22^^,^^37^, ^38^, ^39^, ^40^, ^41^, ^42^, ^43^, ^44^, ^45^, ^46^, ^47^, ^48^, ^49^, ^50^ included (Fig. 1).

### Study characteristics

Overall, the 22 studies included 475 9764 women. Studies were published between 1994 and 2020, with women's age 40 years or older at baseline. Of these studies, seven were done in Asian (3 in China, 1 in Korea, 1 in Japan, 1 in Thailand and 1 in Singapore), six in the USA, eight in Europe (4 in Netherlands, 2 in Sweden, 1 in Italy, and 1 in French), and one contained women from mixed countries (Cuba, Dominican Republic, Puerto Rico and Venezuela, Peru, Mexico and China). Fourteen were case-control studies, six were cohort studies and two were cross-sectional studies (Table 1 and appendix pp 5–20). Numbers of studies on age of menarche and all-cause dementia, AD, VD, CI were 8, 4, 1, 2 respectively. The numbers for age at menopause were 10, 5, 1, 3 respectively; reproductive period were 6, 3, 1, 3 respectively; postmenopausal endogenous estrogen level were 4, 4, 2, 2 respectively. Numbers of studies for each exposure-outcome by types of estimates reported were shown in appendix p 21.

**Table 1:** Characteristics of studies with all-cause dementia by age at menarche, age at menopause, reproductive period and postmenopausal estradiol level.

A) Age of menarche													
Number (author, year)	Categories (year)	Covariates adjusted	Effect sizes, 95% CI					Cases	Control	Person	Age range at baseline (year)	Study type	Country
			OR		RR		HR						
5 # (Rasgon, N. L., 2005)	<12 12–14* >14	Age and education	1.21 (0.85–1.73) 1 1.19 (1.03–1.38)		——		——	42 592 372	192 2834 1444	234 3426 1816	65–84	C	Swedish
18 # (Paganini-Hill, A., 1994)	≤12* 13 ≥14	NA	1 1.14 (0.68–1.52) 1.83 (1.13–2.96)		——		——	32 38 60	175 183 180	207 221 240	86.5 (mean)	Cc	USA
1 # (Geerlings, M. I., 2001)	≤12 13 14 >14*	Age, education, smoking status, alcohol intake, body mass index, hormone replacement therapy, number of children, and apolipoprotein E genotype.	——		1.18 (0.82–1.70) 1.00 (0.67–1.50) 0.89 (0.57–1.29) 1		——	61 40 37 61	——	583 794 672 687	≥55	ir	Netherlands
11 # (Paganini-Hill, A., 2020)	≤12* 13 ≥14	Education	——		——		1 1.22 (0.87–1.72) 0.85 (0.61–1.19)	67 68 74	——	145 130 149	≥90	ci	USA
12 # (Yoo, J. E., 2020)	≤12 13–14* 15–16 ≥17	Age at menarche, age at menopause, parity, duration of breastfeeding, duration of HRT, duration of oral contraceptive use, alcohol consumption, smoking, regular exercise, income, body mass index, hypertension, diabetes mellitus, dyslipidemia and cancer	——		——		1.07 (1.01–1.14) 1 1.07 (1.05–1.09) 1.15 (1.13–1.16)	1134 15,339 70,707 125,047	——	63,275 680,953 1879,203 2073,202	≥40	ir	Korean
13 # (Prince, M. J., 2018)	per year	Age, education and assets	——		——		0.99 (0.94–1.03)	692	——	26,463	≥65	ci	Cuba, Dominican Republic, Puerto Rico and Venezuela, and rural and urban sites in Peru, Mexico and China
19 # (Najar, J., 2019)	per year	Age at menarche, age at menopause, number of pregnancies, months of breastfeeding, birth year, psychological stress, and hypertension	——		——		0.99 (0.91–1.09)	NA	——	1364	38–60	ci	Swedish
20 # (Gilsanz, P.,2018)	≤9 10–13* 14–15 ≥16	Age, race/ethnicity, and educational attainment. Midlife factors include body mass index, hypertension, smoking status. Late life factors include stroke, diabetes, and heart failure, mid and late-life factors.	——		——		1.39 (0.82–2.36) 1 1.00 (0.91–1.11) 1.27 (1.07–1.50)	14 1402 610 341	——	29 3451 1484 789	40–55	ci	USA

B) Age at menopause													
Number (author, year)	Categories (year)	Covariates adjusted		Effect sizes, 95% CI				Cases	Control	Person	Age range at baseline (year)	Study type	Country
				OR	RR	HR							
5 # (Rasgon, N. L., 2005)	<40 40–44 45–49 50–54* >54	Age and education		1.64 (1.03–2.61) 1.39 (1.08–1.78) 1.01 (0.85–1.20) 1 0.96 (0.77–1.19)	——	——		27 102 259 471 134	75 336 1155 2131 689	102 438 1414 2602 823	65–84	C	Swedish
18 # (Paganini-Hill, A., 1994)	≤44* 45–54 ≥55	NA		1 0.96 (0.61–1.52) 1.05 (0.53–2.09)	——	——		34 76 15	139 332 56	173 408 71	86.5 (mean)	Cc	USA
1 # (Geerlings, M. I., 2001)	<48* 48–49 50–52 >52	Age, education, smoking status, alcohol intake, body mass index, hormone replacement therapy, number of children, and apolipoprotein E genotype.		——	1 1.24 (0.72–2.15) 1.95 (1.28–2.96) 1.78 (1.11–2.88)	——		32 23 75 37	——	687 672 794 583	≥55	ci	Netherlands
8 # (Coppus, A. M. W., 2010)	<45 ≥45*	NA		——	——	1.77 (1.10–2.85) 1		37	——	85	≥45	ci	Netherlands
11 # (Paganini-Hill, A., 2020)	≤44* 45–54 ≥55	Education		——	——	1 1.19 (0.84–1.68) 1.13 (0.70–1.82)		43 137 28	——	99 262 59	≥90	ci	USA
12 # (Yoo, J. E., 2020)	<40* 40–44 45–49 50–54 ≥55	Age at menarche, age at menopause, parity, duration of breastfeeding, duration of HRT, duration of oral contraceptive use, alcohol consumption, smoking, regular exercise, income, body mass index, hypertension, diabetes mellitus, dyslipidemia and cancer		——	——	1 0.96 (0.93–0.98) 0.89 (0.86–0.91) 0.85 (0.83–0.87) 0.79 (0.77–0.81)		6308 18,440 59,452 106,193 21,834	——	76,635 248,056 1218,122 2601,970 551,850	≥40	ir	Korean
13 # (Prince, M. J., 2018)	per year	Age, education and assets		——	——	1.00 (0.99–1.01)		692	——	26,463	≥65	ci	Cuba, Dominican Republic, Puerto Rico and Venezuela, and rural and urban sites in Peru, Mexico and China
19 # (Najar, J., 2019)	per year	Age at menarche, age at menopause, number of pregnancies, months of breastfeeding, birth year, psychological stress, and hypertension		——	——	1.07 (1.04–1.10)		NA	——	1364	38–60	ci	Swedish
20 # (Gilsanz, P.,2018)	≤41 42–46 47–49 ≥50*	Age, race/ethnicity, and educational attainment. Midlife factors include body mass index, hypertension, smoking status. Late life factors include stroke, diabetes, and heart failure, mid and late-life factors.		——	——	1.08 (0.96–1.22) 1.06 (0.95–1.19) 0.96 (0.85–1.08) 1		483 561 424 678	——	1219 1376 1050 1495	40–55	ci	USA
9 # (Ryan, J., 2014)	>50* 46–50 41–45 ≤40	Baseline cognitive function, recruitment center, age, education level, physical limitations, chronic illness, depression, use of HT at the menopause and current HT use.		——	——	1 1.23 (0.92–1.64) 1.13 (0.77–1.67) 1.23 (0.76–2.00)		1004 778 175 50	——	1820 1556 366 100	≥65	ci	French

C) Reproductive period													
Number (author, year)	Categories (year)	Covariates adjusted		Effect sizes, 95% CI				Cases	Control	Person	Age range at baseline (year)	Study type	Country
				OR	RR	HR							
5 # (Rasgon, N. L., 2005)	<35 35–39* >39	Age and education		1.15 (0.96–1.36) 1 0.82 (0.66–1.00)	——	——		276 399 157	1131 1911 979	1407 2310 1136	65–84	C	Swedish
1 # (Geerlings, M. I., 2001)	<34* 34–36 37–39 >39	Age, education, smoking status, alcohol intake, body mass index, use of hormone replacement therapy, number of children, and apolipoprotein E genotype.		——	1 1.56 (1.00–2.43) 1.64 (1.07–2.53) 1.78 (1.12–2.84)	——		37 44 50 36	——	687 672 794 583	≥55	ir	Netherlands
11 # (Paganini-Hill, A., 2020)	≤32* 33–38 ≥39	Education		——	——	1 1.06 (0.76–1.47) 0.84 (0.59–1.20)		63 81 64	——	130 150 140	≥90	ci	USA
12 # (Yoo, J. E., 2020)	<30* 30–34 35–39 ≥40	Duration of fertility, parity, duration of breastfeeding, duration of HRT, duration of oral contraceptive use, alcohol consumption, smoking, regular exercise, income, body mass index, hypertension, diabetes mellitus, dyslipidemia and cancer		——	——	1 0.93 (0.92–0.94) 0.81 (0.80–0.82) 0.81 (0.79–0.82)		45,408 97,165 57,242 12,412	——	584,182 1831,593 1916,595 364,263	≥40	ir	Korean
13 # (Prince, M. J., 2018)	per year	Age, education and assets		——	——	1.00 (0.99–1.02)		692	——	26,463	≥65	ci	Cuba, Dominican Republic, Puerto Rico and Venezuela, and rural and urban sites in Peru, Mexico and China
19 # (Najar, J., 2019)	<32.6* 33–35.7 36–37.4 ≥38.0	Reproductive period, number of pregnancies, months of breastfeeding, birth year, exogenous estrogen, physical activity, WHR, hypertension, ischemic heart disease, and psychological stress		——	——	1 1.51 (1.05–2.16) 1.69 (1.17–2.44) 2.17 (1.51–3.11)		53 77 72 88	——	322 343 315 357	38–60	ci	Swedish

D) Estradiol level													
Number (author, year)	Categories	Covariates adjusted		Effect sizes, 95% CI				Cases	Control	Person	Age range at baseline (year)	Study type	Country
				OR	RR	HR							
2 # (Senanarong, V., 2002)	>5 pg/ml * 1.01–5 pg/ml ≤1 pg/ml (Non demented)	NA		——	1 1.13 (0.24–5.46) 6.23 (1.74–22.9)	——		1 1 6	——	17 17 17	68.8(mean)	ci	Thailand
3 # (Geerlings, M. I., 2003)	≥0.0 and <7.1 pmol/L* ≥7.1 and <20 pmol/L ≥20 and ≤67 pmol/L	Age, education, BMI, smoking status, type of menopause, age at natural menopause, and ever use of hormonal replacement therapy.		——	——	1 1.58 (0.75–3.35) 1.99 (0.89–4.45)		22 27 27	——	169 170 169	≥55	ci	Netherlands
7 # (Ravaglia, G., 2007)	Low (undetectable)* High (≥10 pg/mL)	Age, age at menopause, education, apolipoprotein E ε 4 genotype, smoking status, and body mass index, stroke, cardiovascular disease, diabetes, hyperhomocysteinemia, serum folate, serum vitamin B12, and serum creatinine.		——	——	1 1.75 (1.05–2.88)		71	——	433	76.2 (mean)	ci	Italy
11 # (Paganini-Hill, A., 2020)	(EEEI index) ≤32.6* 32.7–35.8 ≥35.9	Education		——	——	1 0.95 (0.68–1.33) 0.77 (0.54–1.08)		71 70 61	——	134 142 135	≥90	ci	USA
23#(Laure Carcaillon.,2014)	Q1: E2 ≤3.49 pg/mL Q2: 3.49–5.30 pg/mL Q3: 5.30–8.00 pg/mL Q4: E2 >8.00 pg/mL	Age and center, education, APOE e4, depressive symptoms, waist-to-hip ratio, Mini-Mental State Examination score at baseline, hypercholesterolemia, and history of myocardial infarction and stroke.		——	——	2.20 (1.07–4.52) 1.46 (0.68–3.15) 1 2.43 (1.15–5.20)		41 26 25 40	——	675	≥65	ci	French

**Notes:** * reference group.

C, Cc, ir and ci represent cross-sectional study, case-control study, person-year cohort study and cumulative number of cases cohort study, respectively.

Most cohort or case-control studies were rated high quality (with scores greater than seven) based on NOS quality assessment tool, and only two studies were rated moderate quality (with a score of six). AHRQ scale for cross-sectional studies also showed that both the two studies were with a high quality (appendix pp 22–23).

### Age at menarche and all-cause dementia, AD, vd and ci

**Figure 2:**
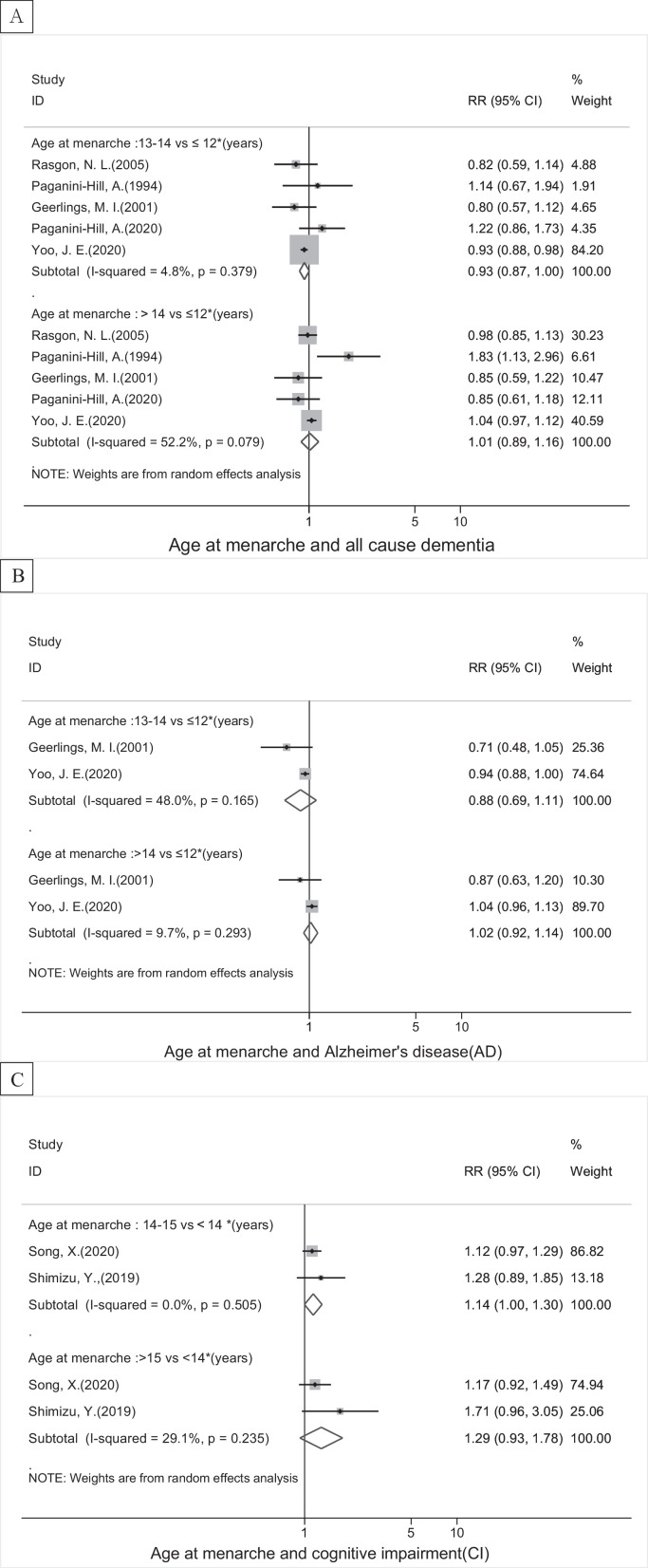
The associations between age at menarche and risk of (A) all-cause dementia, (B) Alzheimer's disease, (C) cognitive impairment. The boxes in the forest plot show the effect estimates from the single studies, and the horizontal lines through the boxes illustrate the width of the 95% confidence interval. The size of each box represents the weight (%) of each study in the meta-analysis. The hollow diamonds show the pooled estimates, and the width of diamond represent the 95% confidence interval.

Compared to women with earlier age at menarche (≤12 years), categorized meta-analysis showed that menarchal age 13–14 years was borderline associated with lower risk of all-cause dementia (pooled RR=0.93, 95% CI 0.87–1.00, I^2^=4.8%), while no clear association with AD (Fig. 2). Later menarche (≥17 years) was related to increased risk of VD (1.16, 1.10–1.22) (Supplementary eTable 2) and a borderline significance was found between later menarchal age and increased risk of and CI (pooled RR=1.14, 95% CI 1.00–1.30, I^2^=0.0%) (Fig. 2). Dose-response meta-analyses showed a J-shape relationship between age at menarche and risk of all-cause dementia and AD. Women with menarche at 13 years had the lowest risk. In addition, a linear relationship was found between menarchal age and risk of CI, and the later of a woman's menarche, the higher risk of experiencing CI (Fig. 5A and Supplementary eFig. 1).

### Age at menopause and all-cause dementia, ad and ci

**Figure 3:**
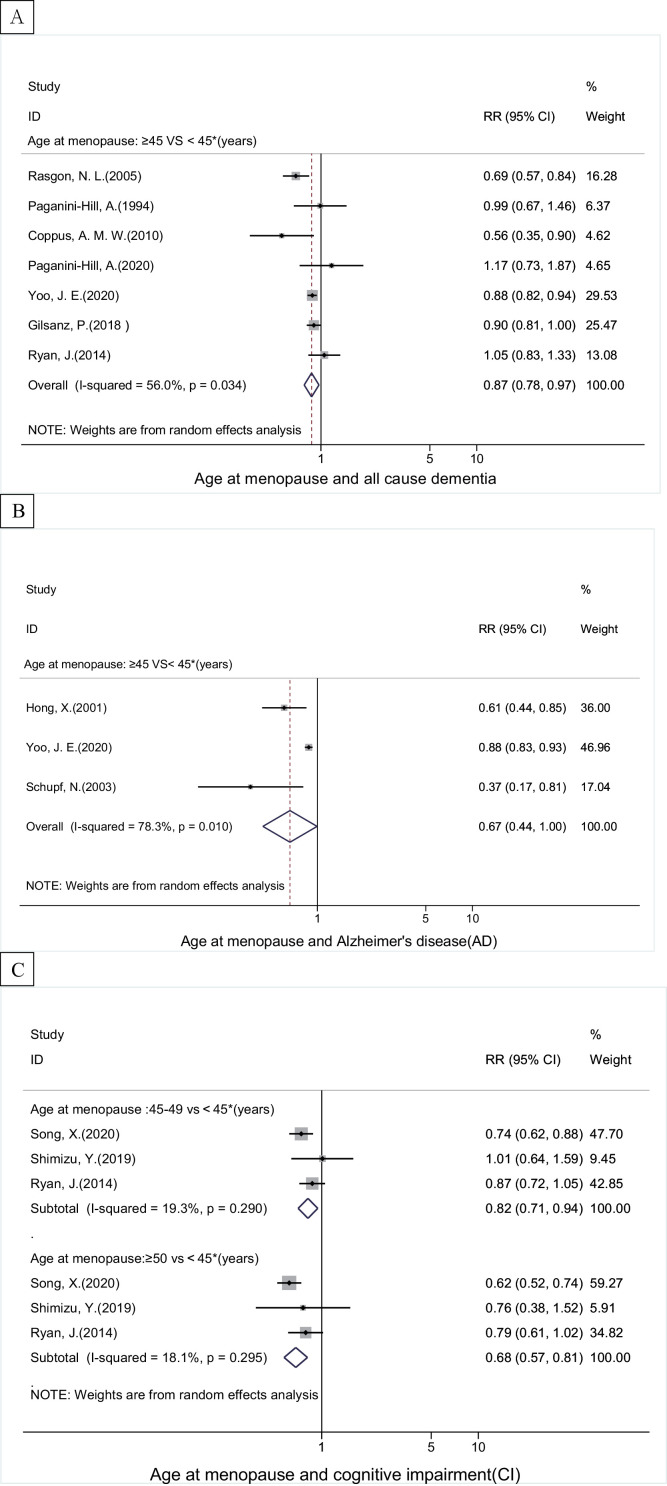
The associations between age at menopause and risk of (A) all-cause dementia, (B) Alzheimer's disease, (C) cognitive impairment. The boxes in the forest plot show the effect estimates from the single studies, and the horizontal lines through the boxes illustrate the width of the 95% confidence interval. The size of each box represents the weight (%) of each study in the meta-analysis. The hollow diamonds show the pooled estimates, and the width of diamond represent the 95% confidence interval.

Compared to women with earlier menopause (<45 years), later menopause (≥45 years) was linked to a decreased risk of all-cause dementia (0.87, 0.78–0.97, I^2^=56.0%), AD (0.67, 0.44–0.99, I^2^=78.3%), VD (0.87, 0.80–0.94) and CI (0.82, 0.71–0.94, I^2^=19.3%) (Fig. 3 and Supplementary eTable 2). Dose-response meta-analyses showed a consistently inverse linear trend, i.e., the later menopausal age, the lower risk of all-cause dementia, AD and CI (Fig. 5B and Supplementary eFig. 1).

### Reproductive period and all-cause dementia, AD, vd and ci

**Figure 4:**
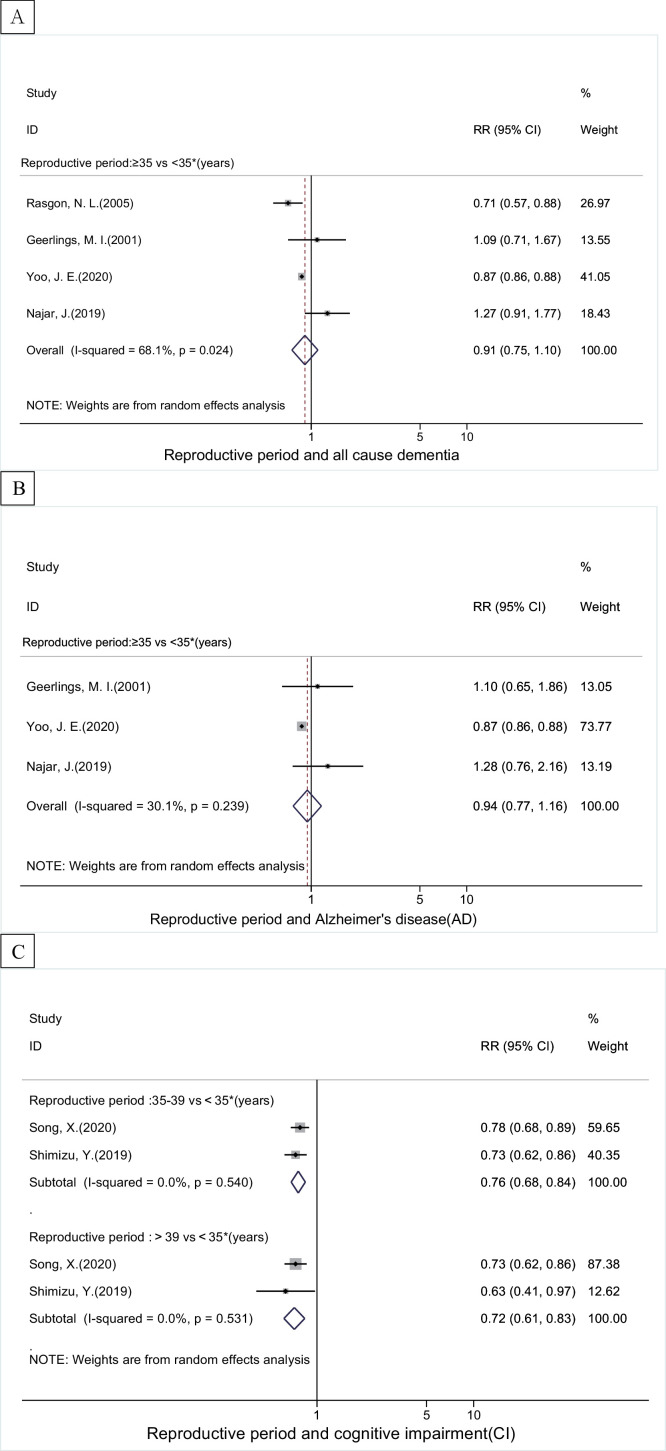
The associations between length of reproductive period and (A) all-cause dementia, (B) Alzheimer's disease, (C) cognitive impairment. The boxes in the forest plot show the effect estimates from the single studies, and the horizontal lines through the boxes illustrate the width of the 95% confidence interval. The size of each box represents the weight (%) of each study in the meta-analysis. The hollow diamonds show the pooled estimates, and the width of diamond represent the 95% confidence interval.

**Figure 5:**
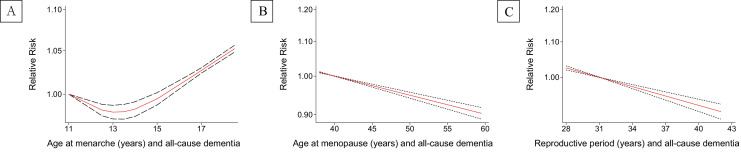
Dose-response meta-analyses for association with all-cause dementia by (A) age at menarche, (B) age at menopause, (C) Reproductive period. The red solid lines represent the estimated dose-response curves and the dashed lines represent the corresponding 95% confidence intervals.

Pooled RRs (95% CI) from categorized meta-analyses showed no association between reproductive duration (≥35 years vs <35 years) and risk of all-cause dementia and AD (Fig. 4), while longer reproductive period (>39 years vs <35 years) was related to lower risk of VD (0.81, 0.76–0.86) and CI (0.72, 0.61–0.83, I^2^=0.0%) (Fig. 4 and Supplementary eTable 2). Dose-response meta-analyses showed an inverse linear relationship between reproductive period and all-cause dementia and CI. A J-shape relationship with AD was also observed, with duration of 37 years had the lowest risk (Fig. 5C and Supplementary eFig. 1).

### Estradiol level and all-cause dementia, AD, vd and ci

An inverse linear association was observed between postmenopausal estradiol concentration and risk of AD and CI (one study with CI also included estradiol level in perimenopausal women). As to the link with all-cause dementia, due to the estradiol levels in available studies were all in a lower level (less then 45 pmol/L), we did not find a clear relationship between them (Supplementary eFig. 1). Although no significance was observed with VD, there was a trend that higher postmenopausal concentration of estradiol might be related to increased risk of VD (Supplementary eTable 2).

### Subgroup analysis

#### By design

Overall, the associations of exposures with outcomes in this study were marginally moderated by study design. Taking age at menopause (≥ 45 vs <45 years) and all-cause dementia for instance, the pooled OR (95% CI) in case-control studies was 0.79 (0.56, 1.12, I^2^=62%), and the pooled RR (95% CI) in cohort studies was 0.89 (0.84, 0.94) (Supplementary eFig. 2).

#### By race

In white population, no significant association was observed between reproductive period and all-cause dementia (RR 95%CI: 0.97, 0.65–1.45, I^2^=0%), while in non-white population, longer reproductive period was linked to lower risk of all-cause dementia (0.87, 0.86–0.88, I^2^=84.5%) (Supplementary eFig. 3).

### Sensitivity analysis

Using the leave-one-out method, we found no studies that influenced the results disproportionately (Supplementary eTable 6). Restriction of the analyses to high-quality studies did not substantially change the pooled estimates (Supplementary eFigure 5). Mantel-Haenszel weighting also yielded similar results (Supplementary eFigure 6).

#### Publication bias

Limited by the number of studies for exposure-outcome relationship, we only conducted Egger test and visualize funnel plot for studies of age at menarche, age at menopause and all-cause dementia. We observed no evidence of publication bias with inspection of the funnel plot or with the Egger test (Supplementary eFigure 7).

## Discussion

Our findings showed that in categorized meta-analyses, later menopausal age was associated with decreased risk of all-cause dementia, AD, VD and CI. Later menarcheal age was linked to higher risk of VD. Longer reproductive duration was related to lower risk of VD and CI. No clear relationship was observed between age at menarche, reproductive duration and risk of AD. In dose-response analyses, there was a J-shape relationship between menarchal age and risk of all-cause dementia and AD, and an inverse linear relationship between menopausal age, reproductive duration, postmenopausal estradiol level and risk of all-cause dementia, AD and CI.

In a systematic review published in 2016, Marios et al. found no association of age at menopause, reproductive duration with dementia and CI.^14^ However, the review did not use uniform classification for age at menopause, and the reference level differed across studies. Thus, interpretation of the pooled estimates was not easy. Original categories of age at menopause or reproductive duration differed in individual studies. Before generating a pooled estimate, it is necessary to reclassify the original classifications into a standardized one.

By using a standard classification of menopausal age, we found later menopause (≥45 years vs <45 years) was associated with decreased risk of all-cause dementia (0.88, 0.78–0.99), AD, VD. Further, when CI was used as an outcome, later menopause and longer reproductive duration (>39 years vs <35 years) were linked to lower risk of CI, consistent with the findings from the previous review.^14^ Another review^51^ found that women with early surgical menopause (≤45 years of age) were associated with a higher risk of all-cause dementia (HR: 1.70, 95%CI: 1.07–2.69) and faster cognitive decline.

Past studies indicated that compared to pre- and perimenopausal women, dramatic decrease in estrogen level after menopause was linked with declined cognitive performance in postmenopausal women.^52^ However, other studies^53^^,^^54^ with direct measurement of endogenous estradiol (total or bioavailable estradiol (i.e., non-SHBG bound)) or estrone, showed inconclusive relationship between endogenous estrogen and cognitive function or dementia. Some studies^7^^,^^25^^,^^55^, ^56^, ^57^ reported protective associations between lifelong endogenous estrogen exposure and cognitive function and many failed to identify any association. Research also showed that endogenous oestradiol level after menopause was linked to cognitive decline. One study found that AD was significantly less frequent among women with the highest levels of postmenopausal oestradiol (oestradiol level range from 5 to 77 pg/mL).^42^ Another study found a u-shape relationship between postmenopausal oestradiol level and risk of all-cause dementia and AD (oestradiol level range from 3.5 to 13 pg/mL).^21^ Several reasons may contribute to the inconsistent findings from previous studies. Studies may collect blood samples in different way, e.g., most studies are based on single blood samples, not always drew fasting or in the early morning.^38^^,^^47^ Also, the component of estradiol measured may differ in studies. Some measured the total estradiol concentration,^22^^,^^41^ while others measured the bioavailable estradiol (i.e., non-SHBG bound).^38^^,^^47^ Consistent with previous studies,^20^^,^^38^ we did not find a clear dose-response relationship between endogenous estradiol (total or bioavailable estradiol) level and all-cause dementia, however a negative association was observed between estradiol and AD, or estradiol and CI, indicating that a higher estradiol level was related to lower risk of AD and CI. Further, taking menopausal hormone therapy (MHT) may affect the estrogen level after menopause. The effect of MHT on dementia depends on types of MHT and timing of use, and may have net harm to other disease. Thus, guidelines recommend against use of MHT for prevention of chronic disease in asymptomatic menopausal women (grade D recommendations).^58^

Several mechanisms have been proposed to explain why lengths and levels of endogenous estrogen exposure affect the cognitive function decline. First, estrogen can remove oxygen free radicals. Estrogen increases the energy production efficiency of mitochondria, thereby inhibiting the mitochondrial production of free radical oxygen molecules.^59^ Thus, loss of estrogens exaggerates aging by decreasing defense against oxidative stress.^60^ Second, endogenous estrogens have protective effects on the cardiovascular system.^61^ Estrogen increases vasodilatation^62^ and inhibits the response of blood vessels to injury and the development of atherosclerosis.^61^ Early loss of estrogen, either natural or surgical menopause may increase expression of inflammatory cytokines and increase the risk of cardiovascular diseases.^63^, ^64^, ^65^ Good vascular health provides adequate blood flow to the brain and benefit central nervous system health. Third, estradiol plays an important role in regulating intracellular Ca^2+^homeostasis and regulating the function of l-type calcium channel, thereby relating to synaptic function and pathological changes of the AD.^66^ In addition, elevated estrogen levels can induce the production of new synapses and dendrites in hippocampal CA1 pyramidal cells.^67^ Fourth, the epsilon 4 allele of the apolipoprotein gene (APOE-ɛ4) is thought to elevate MCI and AD risk partly by increasing neuroinflammation.^68^^,^^69^ Last, estrogen is also related to morphology of the central nervous system. The voxel-based morphometry (VBM) revealed that early menopause might be underlying causes of nervous system degeneration and depression, because it can lead to gray matter volume reduction in certain brain structures.^70^ Also, compared with premenopausal women, there was a significant hippocampal volume reduction bilaterally in postmenopausal women.^71^

Our review has several strengths. Previous reviews^14^^,^^37^^,^^56^ only analyzed the association of premenopausal estrogen exposure (used age at menopause or reproductive duration as indicator) and dementia, and no systematic review has been conducted on the relationship between endogenous estrogen level after menopause and dementia or CI. We included both pre- and postmenopausal estrogen exposure in present review, providing a life-course perspective into understanding the relationship between endogenous estrogen exposure and dementia. Methodologically, besides using categorized meta-analyses to show results as forest plots, we also used dose-response meta-analyses to show the linear/non-linear relationship between the exposure and outcome across a continuous exposure spectrum.

Our review also has several limitations. First, moderate or high heterogeneity among studies was observed when pooled estimates for age at menopause and dementia, or reproductive duration and dementia was calculated. The women's age differed in individual studies, raising the possibility of heterogeneity based on age. Nonetheless, I-square values in this review were in the acceptable range, given the use of random-effects models that account for heterogeneity and yield more conservative effect. In addition, although majorities of studies were adjusted for key potential confounders: education, BMI, smoking and postmenopausal HRT status, other confounding factors affecting lifetime estrogen exposure, such as parity, breastfeeding and oral contraceptive use could not be adjusted in most of the studies. Third, dose-response relationship between postmenopausal estradiol level and CI were from cross-sectional studies and may only reflect a cross-sectional association. Fourth, as to postmenopausal estradiol level, although most included studies detected serum estrogen level using fasting blood at 8–11 am, due to the lack of unified detection methods, the estrogen concentration might fluctuate across studies. Last, limited by the number of studies included, publication bias (using funnel plots) were only assessed for the associations of menopausal age, menarchal age and all-cause dementia.

Later menopause was consistently linked to lower risk of dementia and CI, while menarchal age showed a J-shape relationship with dementia. There was an inverse relationship between higher concentration of postmenopausal estrogen level and risk of AD and CI. Our findings may support the hypothesis that endogenous estrogen loss at menopause confers increased vulnerability to AD in women. Our findings also indicated estrogen exposure indicators before or after menopause might have different effect on VD, with former protective and latter non or harmful. Longitudinal, repeat measure designs are needed to examine the association between life-time estrogen exposure and risk of subtypes of dementia using direct measure of serum level of endogenous estrogen before and after menopause.

## Funding

Start-up Foundation for Scientific Research in Shandong University.

## Contributors

CF and WH searched databases, extracted data, did statistical analyses, and drafted the manuscript. NS, SSV, SRM, and DZ contributed to critical revision of the manuscript. DZ was the study supervisor and conceived the study design and contributed to interpretation of the results.

## Declaration of interest

Dr. Virani reports grants from Department of Veterans Affairs, NIH, World Heart Federation, Tahir and Jooma Family, other from being an associate editor for Innovations of American College of Cardiology(acc.org), outside the submitted work. All the other authors report no conflicts.

## Supplementary materials

Image, application 1
