# Supplementary material for: Association of reproductive factors with dementia: A systematic review and dose-response meta-analyses of observational studies
Source: eClinicalMedicine. 2021 Dec 14;43:101236. doi: 10.1016/j.eclinm.2021.101236 (PMC8683685; doi:10.1016/j.eclinm.2021.101236)
Supplement: Supplementary file 1 [file mmc1.docx]

**Supplementary Online Content**

Fu CY, Hao WT, Wang YX, Liu KK, Hou TT, Zhang QH, Shrestha N, Virani SS, Mishra SR, Du YF, Zhu DS. Association of reproductive factors with dementia: systematic review and dose-response meta-analyses of observational studies.

**eMethods.** Search Strategies

**eTable 1.** Characteristics of studies with Alzheimer's disease (AD) by age at menarche, age at menopause, reproductive period and postmenopausal estradiol level

**eTable 2.** Characteristics of studies with vascular dementia (VD) by age at menarche, age at menopause, reproductive period and postmenopausal estradiol level

**eTable 3.** Characteristics of studies with cognitive impairment (CI) by age at menarche, age at menopause, reproductive period and postmenopausal estradiol level

**eTable 4.** Numbers of studies for each exposure-outcome by types of estimates reported

**eTable 5.** Quality Assessment According to the Newcastle-Ottawa Scale and AHRQ evaluation scale

**eTable 6.** Sensitivity Analysis by the Leaving-One-Out Method

**eTable 7.** List of studies excluded at full-text screening stage

**eTable 8.** Meta-analysis of Observational Studies in Epidemiology (MOOSE) Checklist

**eFigure 1.** Dose-response meta-analyses for the relationship between reproductive factors and Alzheimer's disease (AD) and cognitive impairment (CI)

**eFigure 2.** Subgroup Analysis by Design

**eFigure 3.** Subgroup Analysis by Race

**eFigure 4.** Subgroup Analysis by Age

**eFigure 5.** Sensitivity Analysis of Studies With High Quality

**eFigure 6.** Sensitivity Analyses by Mantel-Haenszel Weighting

**eFigure 7.** Funnel Plot and Egger’s test.

**e Methods. Search Strategies**

1. **Search strategy for PubMed (July 24, 2021)**

| **#** | **Searches** | **Results** |
| --- | --- | --- |
| #1 | "age at menarche"[Title/Abstract] OR "menarch*"[Title/Abstract] OR "pubert*"[Title/Abstract] OR "sexual maturation"[Title/Abstract] OR "precocious"[Title/Abstract] OR "menarche"[MeSH Terms] OR "puberty"[MeSH Terms] OR "sexual maturation"[MeSH Terms] | 67,037 |
| #2 | "age at menopause"[Title/Abstract] OR "menopaus*"[Title/Abstract] OR "climacteric"[Title/Abstract] OR "perimenopaus*"[Title/Abstract] OR "postmenopaus*"[Title/Abstract] OR "onset menopause"[Title/Abstract] OR "age at natural menopause"[Title/Abstract] OR "final menstrual period"[Title/Abstract] OR "final menstruation"[Title/Abstract] OR "menopause"[MeSH Terms] OR "menopause"[MeSH Terms] OR "climacteric"[MeSH Terms] OR "perimenopause"[MeSH Terms] OR "postmenopause"[MeSH Terms] | 111,364 |
| #3 | "reproductive timing"[Title/Abstract] OR "reproductive time"[Title/Abstract] OR "reproductive duration"[Title/Abstract] OR "reproductive year*"[Title/Abstract] OR "reproductive history"[Title/Abstract] OR "reproductive span*"[Title/Abstract] OR "reproductive life span*"[Title/Abstract] OR "Reproductive period"[Title/Abstract] | 5,355 |
| #4 | "Estrogen exposure"[Title/Abstract] OR "Estrogen index"[Title/Abstract] OR "Oestrogen exposure"[Title/Abstract] OR "Oestrogen index"[Title/Abstract] | 1,195 |
| #5 | "Dementia"[Title/Abstract] OR "cognitive capabilit*"[Title/Abstract] OR "Alzheimer's disease"[Title/Abstract] OR "Cognitive impairment"[Title/Abstract] OR "Cognition function"[Title/Abstract] OR "cognitive function"[Title/Abstract] OR "Alzheimer"[Title/Abstract] | 285,684 |
| #6 | #1 OR #2 OR #3 OR #4 AND #5 | 1,972 |

1. **Search strategy for Embase (July 24, 2021)**

| **#** | **Searches** | **Results** |
| --- | --- | --- |
| #1 | 'age at menarche':ab,ti OR menarch*:ab,ti OR pubert*:ab,ti OR 'sexual maturation':ab,ti OR  precocious:ab,ti | 78,755 |
| #2 | 'age at menopause':ab,ti OR menopaus*:ab,ti OR climacteric:ab,ti OR perimenopaus*:ab,ti OR postmenopaus*:ab,ti OR 'onset menopause':ab,ti OR 'age at natural menopause':ab,ti OR 'final menstrual period':ab,ti OR 'final menstruation':ab,ti | 144,037 |
| #3 | 'reproductive timing':ab,ti OR 'reproductive time':ab,ti OR 'reproductive duration':ab,ti OR 'reproductive year*':ab,ti OR 'reproductive history':ab,ti OR 'reproductive span*':ab,ti OR 'reproductive life span*':ab,ti OR 'reproductive period':ab,ti | 6,806 |
| #4 | 'estrogen exposure':ab,ti OR 'estrogen index':ab,ti OR 'oestrogen exposure':ab,ti OR 'oestrogen index':ab,ti | 1,573 |
| #5 | dementia:ab,ti OR 'cognitive capabilit*':ab,ti OR 'alzheimer disease':ab,ti OR 'cognitive impairment':ab,ti OR 'cognition function':ab,ti OR 'cognitive function':ab,ti OR alzheimer:ab,ti | 396,143 |
| #6 | #1 OR #2 OR #3 OR #4 AND #5 | 2,702 |

1. **Search strategy for Web of Science Core Collection (July 24, 2021)**

| **#** | **Searches** | **Results** |
| --- | --- | --- |
| #1 | TOPIC: ("age at menarche") OR TOPIC: (menarch*) OR TOPIC: (pubert*) OR   TOPIC: ("sexual maturation") OR TOPIC: (precocious) | 54,346 |
| #2 | TOPIC: ("age at menopause") OR TOPIC: (menopaus*) OR TOPIC: (climacteric) OR TOPIC: (perimenopaus*) OR TOPIC: (postmenopaus*) OR TOPIC: ("onset menopause") OR TOPIC: ("age at natural menopause") OR TOPIC: ("final menstrual period") OR TOPIC: ("final menstruation") | 124,383 |
| #3 | TOPIC: ("reproductive timing") OR TOPIC: ("reproductive time") OR TOPIC:  ("reproductive duration") OR TOPIC: ("reproductive year*") OR TOPIC: ("reproductive history") OR TOPIC: ("reproductive span*") OR TOPIC: ("reproductive life span*") OR TOPIC: ("Reproductive period") | 7,095 |
| #4 | TOPIC: ("Estrogen* exposure") OR TOPIC: ("Estrogen* index") OR TOPIC: ("Oestrogen* exposure") OR TOPIC: ("Oestrogen* index") | 1,437 |
| #5 | TOPIC: (Dementia) OR TOPIC: ("Cognitive capabilit*") OR TOPIC: ("Alzheimer's disease") OR TOPIC: ("Cognitive impairment ") OR TOPIC: ("Cognition function") OR TOPIC: ("cognitive function") OR TOPIC: (Alzheimer) | 361,415 |
| #6 | #1 OR #2 OR #3 OR #4 AND #5 | 3,183 |

**e Table 1. Characteristics of studies with Alzheimer's disease (AD) by age at menarche, age at menopause, reproductive period and postmenopausal estradiol level**

| **A) Age of menarche** | | | | | | | | | | | | |
| --- | --- | --- | --- | --- | --- | --- | --- | --- | --- | --- | --- | --- |
| **Number (author, year)** | **Categories (year)** | **Covariates adjusted** | **Effect sizes, 95% CI** | | | **Cases** | **Control** | **Person** | **Age range at baseline (year)** | **Study type** | **Country** | |
|  |  |  | **OR** | **RR** | **HR** |  |  |  |  |  |  |  |
| 15 # (Hong, X., 2001) | per year | Age at menarche, age at natural menopause, number of children breastfeeding, education level, occupation, residence status, average annual income, history of brain injury | 1.16 (1.03-1.31) | —— | —— | 115 | 1,041 | 1,156 | ≥55 | Cc | China | |
| 1 # (Geerlings, M. I., 2001) | ≤12 13 14 ＞14* | Age, education, smoking status, alcohol intake, body mass index, hormone replacement therapy, number of children, and apolipoprotein E genotype. | —— | 1.15 (0.77-1.72) 0.89 (0.56-1.40) 0.76 (0.48-1.21) 1 | —— | 49 30 28 52 | —— | 583 794 672 687 | ≥55 | ci | Netherlands | |
| 12 # (Yoo, J. E., 2020) | ≤12 13-14* 15-16 ≥17 | Age at menarche, age at menopause, parity, duration of breastfeeding, duration of HRT, duration of oral contraceptive use, alcohol consumption, smoking, regular exercise, income, body mass index, hypertension, diabetes mellitus, dyslipidemia and cancer | —— | —— | 1.06 (0.99-1.14) 1 1.06 (1.04-1.09) 1.14 (1.12-1.16) | 848 11,663 54,154 96,236 | —— | 63,275 680,953 1,879,203 2,073,202 | ≥40 | ir | Korean | |
| 19 # (Najar, J., 2019) | per year | Age at menarche, age at menopause, number of pregnancies, months of breastfeeding, birth year, psychological stress, and hypertension | —— | —— | 1.01 (0.89-1.15) | NA | —— | 1,364 | 38-60 | ci | Swedish | |
| **B) Age at menopause** | | | | | | | | | | | | |
| **Number (author, year)** | **Categories (year)** | **Covariates adjusted** | **Effect sizes, 95% CI** | | | **Cases** | **Control** | **Person** | **Age range at baseline (year)** | **Study type** | | **Country** |
|  |  |  | **OR** | **RR** | **HR** |  |  |  |  |  |  |  |
| 15 # (Hong, X., 2001) | ＜47* 47-50 ＞50 | Age at menarche, age at natural menopause, number of children breastfeeding, education level, occupation, residence status, average annual income, history of brain injury | 1 0.67 (0.48-0.92) 0.45 (0.23-0.85) | —— | —— | 36 43 11 | 296 392 278 | 332 435 289 | ≥55 | Cc | | China |
| 1 # (Geerlings, M. I., 2001) | ＜48* 48-49 50-52 ＞52 | Age, education, smoking status, alcohol intake, body mass index, hormone replacement therapy, number of children, and apolipoprotein E genotype. | —— | 1 0.90 (0.48-1.68) 1.64 (1.05-2.56) 1.47 (0.88-2.46) | —— | 30 16 60 29 | —— | 687 672 794 583 | ≥55 | ci | | Netherlands |
| 12 # (Yoo, J. E., 2020) | <40* 40-44 45-49 50-54 ≥55 | Age at menarche, age at menopause, parity, duration of breastfeeding, duration of HRT, duration of oral contraceptive use, alcohol consumption, smoking, regular exercise, income, body mass index, hypertension, diabetes mellitus, dyslipidemia and cancer | —— | —— | 1 0.96 (0.93-0.99) 0.88 (0.86-0.91)  0.85 (0.82-0.87) 0.79 (0.77-0.82) | 4,893 14,330 45,753 81,274 16,651 | —— | 76,635 248,056 1,218,122 2,601,970 551,850 | ≥40 | ir | | Korean |
| 19 # (Najar, J., 2019) | per year | Age at menarche, age at menopause, number of pregnancies, months of breastfeeding, birth year, psychological stress, and hypertension | —— | —— | 1.07 (1.02-1.12) | NA | —— | 1,364 | 38-60 | ci | | Swedish |
| 4 # (Schupf, N., 2003) | ≤46 ＞46* | Age, level of mental retardation, body mass index, and history of hypothyroidism or depression | —— | 2.70 (1.20-5.90) | —— | 18 12 | —— | 65 46 | 40-60 | ci | | USA |
| **C) Reproductive period** | | | | | | | | | | | | |
| **Number (author, year)** | **Categories (year)** | **Covariates adjusted** | **Effect sizes, 95% CI** | | | **Cases** | **Control** | **Person** | **Age range at baseline (year)** | **Study type** | | **Country** |
|  |  |  | **OR** | **RR** | **HR** |  |  |  |  |  |  |  |
| 1 # (Geerlings, M. I., 2001) | ＜34* 34-36 37-39 ＞39 | Age, education, smoking status, alcohol intake, body mass index, use of hormone replacement therapy, number of children, and apolipoprotein E genotype. CI indicates confidence interval. | —— | 1 1.32 (0.81-2.14) 1.41 (0.88-2.25) 1.51 (0.91-2.50) | —— | 34 34 39 28 | —— | 687 672 794 583 | ≥55 | ci | | Netherlands |
| 12 # (Yoo, J. E., 2020) | ＜30* 30-34 35-39 ≥40 | Duration of fertility, parity, duration of breastfeeding, duration of HRT, duration of oral contraceptive use, alcohol consumption, smoking, regular exercise, income, body mass index, hypertension, diabetes mellitus, dyslipidemia and cancer | —— | —— | 1 0.93 (0.92-0.94) 0.81 (0.80-0.82) 0.81 (0.79-0.83) | 35,210 74,555 43,690 9,446 | —— | 584,182 1,831,593 1,916,595 364,263 | ≥40 | ir | | Korean |
| 19 # (Najar, J., 2019) | ＜32.6* 33-35.7 36-37.4 ≥38.0 | Reproductive period, number of pregnancies, months of breastfeeding, birth year, exogenous estrogen, physical activity, WHR, hypertension, ischemic heart disease, and psychological stress | —— | —— | 1 1.81 (1.07-3.07) 1.91 (1.11-3.28) 2.78 (1.65-4.71) | 23 40 35 47 | —— | 322 343 315 357 | 38-60 | ci | | Swedish |

| **D) Estradiol level** | | | | | | | | | | | | |
| --- | --- | --- | --- | --- | --- | --- | --- | --- | --- | --- | --- | --- |
| **Number (author, year)** | **Categories** | **Covariates adjusted** | **Effect sizes, 95% CI** | | | | **Cases** | **Control** | **Person** | **Age range at baseline (year)** | **Study type** | **Country** |
|  |  |  | **OR** | | **RR** | **HR** |  |  |  |  |  |  |
| 21 # (Manly, J. J., 2000) | 19.9–77.0 pg/mL* 16.0–19.8 pg/mL 12.0–15.9 pg/mL 5.0–11.9 pg/mL | Age, years of education, ethnicity, body mass index, and presence of APOE-ε4 allele. | 1  3.20 (0.90-11.70) 3.90 (1.00-15.40) 4.20 (1.10-15.60) | | —— | —— | 5 16 12 17 | —— | 36 39 33 35 | 75.4 (mean) | Cc | USA |
| 3 # (Geerlings, M. I., 2003) | ≥0.0 and ＜7.1 pmol/L* ≥7.1 and ＜20 pmol/L ≥20 and ≤67 pmol/L | Age, education, BMI, smoking status, type of menopause, age at natural menopause, and ever use of hormonal replacement therapy. | —— | | —— | 1  1.58 (0.70-3.55)  1.41 (0.57-3.50) | 17 22 16 | —— | 169 170 169 | ≥55 | ci | Netherlands |
| 7 # (Ravaglia, G., 2007) | Low (undetectable)* High (>10 pg/mL) | Age, age at menopause, education, apolipoprotein E ε 4 genotype, smoking status, and body mass index, stroke, cardiovascular disease, diabetes, hyperhomocysteinemia, serum folate, serum vitamin B12, and serum creatinine. | | —— | —— | 1  1.94 (1.04-3.60) | 46 | —— | 433 | 76.2 (mean) | ci | Italy |
| 6 # (Schupf, N., 2006) | **Bioavailable estradiol:** > 8.8 pg/ml* ≤8.8 pg/ml | Age, level of mental retardation, ethnicity body mass index, history of hypothyroidism or depression, and the presence of the APOE 4 allele. | —— | | —— | 1  4.10 (1.20-13.90) | 17 | —— | 96 | 42-59 | ci | USA |
| 23 # (Carcaillon, L.,2014) | Q1: E2 ≤3.49 pg/mL  Q2: 3.49–5.30 pg/mL  Q3: 5.30–8.00 pg/mL  Q4: E2 >8.00 pg/mL | Age and center, education, APOE e4, depressive symptoms, waist-to-hip ratio, Mini-Mental State Examination score at baseline, hypercholesterolemia, and history of myocardial infarction and stroke | —— | | —— | 2.07 (0.89–4.75)  1.37 (0.57–3.35)  1  2.38 (0.99–5.75) | 28  18  17  27 | —— | 675 | ≥65 | ci | French |
| **Notes:** * reference group. Cc, ir and ci represent case-control study, person-year cohort study and cumulative number of cases cohort study, respectively. | | | | | | | | | | | | |

**e Table 2. Characteristics of studies with vascular dementia (VD) by age at menarche, age at menopause, reproductive period and postmenopausal estradiol level**

| **A) Age of menarche** | | | | | | | | | | | |
| --- | --- | --- | --- | --- | --- | --- | --- | --- | --- | --- | --- |
| **Number (author, year)** | **Age at menarche (year)** | **Covariates adjusted** | **Effect sizes, 95% CI** | | | **Cases** | **Control** | **Person** | **Age range at baseline (year)** | **Study type** | **Country** |
|  |  |  | **OR** | **RR** | **HR** |  |  |  |  |  |  |
| 12 # (Yoo, J. E., 2020) | ≤12, 13-14*, 15-16, ≥17 | Age at menarche, age at menopause, parity, duration of breastfeeding, duration of HRT, duration of oral contraceptive use, alcohol consumption, smoking, regular exercise, income, body mass index, hypertension, diabetes mellitus, dyslipidemia and cancer | —— | —— | 1.14 (0.96, 1.34) 1 1.08 (1.03, 1.13) 1.16 (1.10, 1.22) | 146 1,825 8,123 13,935 | —— | 63,275 680,953 1,879,203 2,073,202 | ≥40 | ir | Korean |
| **B) Age at menopause** | | | | | | | | | | | |
| **Number (author, year)** | **Age at menopause (year)** | **Covariates adjusted** | **Effect sizes, 95% CI** | | | **Cases** | **Control** | **Person** | **Age range at baseline (year)** | **Study type** | **Country** |
|  |  |  | **OR** | **RR** | **HR** |  |  |  |  |  |  |
| 12 # (Yoo, J. E., 2020) | <40*, 40-44, 45-49, 50-54, ≥55 | Age at menarche, age at menopause, parity, duration of breastfeeding, duration of HRT, duration of oral contraceptive use, alcohol consumption, smoking, regular exercise, income, body mass index, hypertension, diabetes mellitus, dyslipidemia and cancer | —— | —— | 1 0.91 (0.83, 0.99) 0.87 (0.80, 0.94) 0.83 (0.77, 0.90) 0.76 (0.70, 0.83) | 706 1,949 6,666 12,158 2,550 | —— | 76,635 248,056 1,218,122 2,601,970 551,850 | ≥40 | ir | Korean |

| **C) Reproductive period** | | | | | | | | | | | | | | | |  |
| --- | --- | --- | --- | --- | --- | --- | --- | --- | --- | --- | --- | --- | --- | --- | --- | --- |
| **Number (author, year)** | **Reproductive period (year)** | | **Covariates adjusted** | **Effect sizes, 95% CI** | | | | **Cases** | | **Control** | **Person** | **Age range at baseline (year)** | | **Study type** | **Country** |  |
|  |  |  |  | **OR** | **RR** | **HR** | |  |  |  |  |  |  |  |  |  |
| 12 # (Yoo, J. E., 2020) | ＜30*, 30-34, 35-39, ≥40 | | Duration of fertility, parity, duration of breastfeeding, duration of HRT, duration of oral contraceptive use, alcohol consumption, smoking, regular exercise, income, body mass index, hypertension, diabetes mellitus, dyslipidemia and cancer | —— | —— | 1 0.95 (0.92, 0.98) 0.82 (0.79, 0.85) 0.81 (0.76, 0.86) | | 4,874 10,909 6,773 1,473 | | —— | 584,182 1,831,593 1,916,595  364,263 | ≥40 | | ir | Korean |  |
| **D) Estradiol level** | | | | | | | | | | | | | | | |  |
| **Number (author, year)** | | **Estradiol level** | **Covariates adjusted** | **Effect sizes, 95% CI** | | | | | **Cases** | **Control** | **Person** | **Age range at baseline (year)** | **Study type** | | **Country** |  |
|  |  |  |  | **OR** | **RR** | | **HR** | |  |  |  |  |  |  |  |  |
| 3 # (Geerlings, M. I., 2003) | | ≥0.0 and ＜7.1 pmol/L*， ≥7.1 and ＜20 pmol/L， ≥20 and ≤67 pmol/L | Age, education, BMI, smoking status, type of menopause, age at natural menopause, and ever use of hormonal replacement therapy. | —— | —— | | 1 0.76 (0.12-4.92) 3.68 (0.92-14.76) | | 3 2 7 | —— | 169 170 169 | ≥55 | ci | | Netherlands |  |
| 7 # (Ravaglia, G., 2007) | | Low (undetectable)*, High (≥10 pg/mL) | Age, age at menopause, education, apolipoprotein E ε 4 genotype, smoking status, and body mass index, stroke, cardiovascular disease, diabetes, hyperhomocysteinemia, serum folate, serum vitamin B12, and serum creatinine. | —— | —— | | 1 1.74 (0.61-4.40） | | 21 | —— | 433 | 76.2 (mean) | ci | | Italy | |
| **Notes**: * reference group. Cc, ir and ci represent case-control study, person-year cohort study and cumulative number of cases cohort study, respectively. | | | | | | | | | | | | | | | |  |

**e Table 3. Characteristics of studies with cognitive impairment (CI) by age at menarche, age at menopause, reproductive period and postmenopausal estradiol level**

| **A) Age of menarche** | | | | | | | | | | | | | | |
| --- | --- | --- | --- | --- | --- | --- | --- | --- | --- | --- | --- | --- | --- | --- |
| **Number (author, year)** | **Categories (year)** | **Cognitive assessment** | **Covariates adjusted** | **Effect sizes, 95% CI** | | | | | **Cases** | **Control** | **Person** | **Age range at baseline (year)** | **Study type** | **Country** |
|  |  |  |  | **OR** | **RR** | | **HR** | |  |  |  |  |  |  |
| 16 # (Song, X., 2020) | <13* 13-14 15-16 >16 Per year; | MMSE. Education-specific cut-off points. 17/18 for subjects with no formal education, 20/21 for subjects with primary school education, and 24/25 for those with secondary school or higher education. | Age at Mini-Mental State Examination measurement, year of baseline interview, dialect group, marital status, and education level, smoking status, tea intake, coffee intake, sleep duration, physical activity, body mass index, total energy intake, alternate Mediterranean dietary pattern score, baseline history of hypertension, diabetes, cardiovascular disease, and cancer. | 1 1.02 (0.86-1.22) 1.13 (0.94-1.36) 1.18 (0.93-1.49) 1.04 (1.00-1.07) | —— | | —— | | 260 459 430 183 | —— | 2,034 3,046 2,325 817 | 45-74 | ci | Singapore Chinese |
| 17 # (Shimizu, Y., 2019) | ≤13* 14-15 ≥16 | MMSE. MMSE cut-off point for dementia was set at 23. | Age at examination(continuous), BMI (< 21.8, 21.8–24.1, ≥24.1 kg/m2), educational background (junior high school, high school or higher), smoking (never, ever), leisure-time physical exercise (less than one day/week, one day/week or more), and past medical history (none or any of hypertension, diabetes mellitus, or depression) | 1 1.28 (0.87-1.89) 1.70 (0.96-2.99) | —— | | —— | | 63 113 46 | —— | 238 320 92 | 40-79 | ci | Japan |
| **B) Age at menopause** | | | | | | | | | | | | | | |
| **Number (author, year)** | **Categories (year)** | **Cognitive assessment** | **Covariates adjusted** | **Effect sizes, 95% CI** | | | | | **Cases** | **Control** | **Person** | **Age range at baseline (year)** | **Study type** | **Country** |
|  |  |  |  | **OR** | | **RR** | | **HR** |  |  |  |  |  |  |
| 16 # (Song, X., 2020) | <45 45-49 50-54* >54 Per year | MMSE. Education-specific cut-off points. 17/18 for subjects with no formal education, 20/21 for subjects with primary school education, and 24/25 for those with secondary school or higher education. | Age at Mini-Mental State Examination measurement, year of baseline interview, dialect group, marital status, and education level, smoking status, tea intake, coffee intake, sleep duration, physical activity, body mass index, total energy intake, alternate Mediterranean dietary pattern score, baseline history of hypertension, diabetes, cardiovascular disease, and cancer. | 1.67 (1.32-2.11) 1.24 (1.08-1.44) 1 1.06 (0.87-1.29) 0.97 (0.96-0.99) | | —— | | —— | 131 419 600 182 | —— | 508 2,291 4,361 1,062 | 45-74 | ci | Singapore Chinese |
| 17 # (Shimizu, Y., 2019) | ≤44* 45-49 ≥50 | MMSE. MMSE cut-off point for dementia was set at 23. | Age at examination(continuous), BMI (< 21.8, 21.8–24.1, ≥24.1 kg/m2), educational background (junior high school, high school or higher), smoking(never, ever), leisure-time physical exercise (less than one day/week, one day/week or more), and past medical history (none or any of hypertension, diabetes mellitus, or depression) | 1 1.01 (0.55-1.86) 0.76 (0.43-1.36) | | —— | | —— | 23 76 117 | —— | 58 192 341 | 40-79 | ci | Japan |
| 9 # (Ryan, J., 2014) | ＞50* 46-50 41-45 ≤40 | Global function MMSE < 26 | Recruitment centre, age, education level, physical limitations, chronic illness, depression, use of HT at the menopause and current HT use | 1 1.10 (0.91-1.32) 1.21 (0.90-1.63) 1.44 (0.88-2.37) | | —— | | —— | 1,074 884 199 50 | —— | 1,820 1,556 366 100 | ≥65 | ci | French |
|  |  |  |  |  |  |  |  |  |  |  |  |  |  |  |

| **C) Reproductive period** | | | | | | | | | | | | |
| --- | --- | --- | --- | --- | --- | --- | --- | --- | --- | --- | --- | --- |
| **Number (author, year)** | **Categories**  **(year)** | **Cognitive assessment** | **Covariates adjusted** | **Effect sizes, 95% CI** | | | **Cases** | **Control** | **Person** | **Age range at baseline (year)** | **Study type** | **Country** |
|  |  |  |  | **OR** | **RR** | **HR** |  |  |  |  |  |  |
| 10 # (Li, F. D., 2016) | per year | MMSE. Education-specific cut-off points.17/18 for illiteracy, 20/21 for people with primary education level, 24/25 for people with higher  than primary education level. | Age, race, education, marital status, economic status, smoking, alcohol drinking, exercise, hypertension and depressive symptom | 0.97 (0.96, 1.00) | —— | —— | 919 |  | 4,751 | ≥65 | ci | China |
| 16 # (Song, X., 2020) | <35 35-39* >39 Per year | MMSE. Education-specific cut-off points. 17/18 for subjects with no formal education, 20/21 for subjects with primary school education, and 24/25 for those with secondary school or higher education. | Age at Mini-Mental State Examination measurement, year of baseline interview, dialect group, marital status, and education level, smoking status, tea intake, coffee intake, sleep duration, physical activity, body mass index, total energy intake, alternate Mediterranean dietary pattern score, baseline history of hypertension, diabetes, cardiovascular disease, and cancer. | 1.28 (1.11-1.48) 1 0.94 (0.79-1.11) 0.97 (0.96-0.98) | —— | —— | 477 604 251 | —— | 2,193 4,155 1,874 | 45-74 | ci | Singapore Chinese |
| 17 # (Shimizu, Y., 2019) | ≤33* 34-37 ≥38 | MMSE. MMSE cut-off point for dementia was set at 23. | Age at examination(continuous), BMI(< 21.8, 21.8–24.1, ≥24.1 kg/m2), educational background (junior high school, high school or higher), smoking(never, ever), leisure-time physical exercise (less than one day/week, one day/week or more), and past medical history (none or any of hypertension, diabetes mellitus, or depression) | 1 0.89 (0.58-1.36) 0.63 (0.41-0.96) | —— | —— | 73 75 63 | —— | 171 191 210 | 40-79 | ci | Japan |

| **D) Estradiol level** | | | | | | | | | | | | |
| --- | --- | --- | --- | --- | --- | --- | --- | --- | --- | --- | --- | --- |
| **Number (author, year)** | **Categories** | **Cognitive assessment** | **Covariates adjusted** | **Effect sizes, 95% CI** | | | **Cases** | **Control** | **Person** | **Age range at baseline (year)** | **Study type** | **Country** |
|  |  |  |  | **OR** | **RR** | **HR** |  |  |  |  |  |  |
| 14 # (Lebrun, C. E., 2005) | 0–10 pmol/l* 10–15 pmol/l 15–20 pmol/l 20–29 pmol/l 29–102 pmol/l | Mild cognitive impairment (MMSE < 27),  moderate cognitive impairment (MMSE < 26) | Age, MAP, SHBG, BMI, educational level | 1  1.30 (0.65-2.60) 0.93 (0.58-2.23) 0.72 (0.35-1.49) 0.50 (0.23-1.08) | —— | —— | 32 35 36 25 21 | —— | 83 87 87 74 80 | 50-74 | C | Netherlands |
| 22 # (Hu, J., 2017) | 0–20.97 pmol/L* 20.98–51.38 pmol/L 51.39–129.50 pmol/L 129.51- pmol/L | MoCA＜26 | Age, educational level and other potential confounders were adjusted | 1  0.22(0.072-0.67)  0.012(0.003-0.052) 0.019(0.004-0.087) | —— | —— | 43 29 4 6 | 7 21 46 43 | 50 50 50 49 | 40-65 | C | China |
| **Notes:** * reference group. Cc, ir and ci represent case-control study, person-year cohort study and cumulative number of cases cohort study, respectively. C represent cross-sectional study. | | | | | | | | | | | | |

| **e Table 4. Numbers of studies for each exposure-outcome by types of estimates reported** | | | | | | |
| --- | --- | --- | --- | --- | --- | --- |
|  | |  | **All-cause dementia** | **Alzheimer's disease (AD)** | **Vascular dementia (VD)** | **Cognitive impairment (CI)** |
| Menarche | OR | | 2 | 1 | 0 | 2 |
|  | RR | | 1 | 1 | 0 | 0 |
|  | HR | | 5 | 2 | 1 | 0 |
| Menopause | OR | | 2 | 1 | 0 | 3 |
|  | RR | | 1 | 2 | 0 | 0 |
|  | HR | | 7 | 2 | 1 | 0 |
| Reproductive  duration | OR | | 1 | 0 | 0 | 3 |
|  | RR | | 1 | 1 | 0 | 0 |
|  | HR | | 4 | 2 | 1 | 0 |
| Estradiol  level | OR | | 0 | 1 | 0 | 2 |
|  | RR | | 1 | 0 | 0 | 0 |
|  | HR | | 3 | 3 | 2 | 0 |
| OR, odds ratio; RR, relative risk or rate ratio; HR, hazard ratio | | | | | | |

**e Table 5. Quality Assessment According to the Newcastle-Ottawa Scale and AHRQ evaluation scale**

| **A) NOS for case-control study and cohort study** | | | | | | | | | | | | | | | |
| --- | --- | --- | --- | --- | --- | --- | --- | --- | --- | --- | --- | --- | --- | --- | --- |
| **Author** | **Publication year** | **Study type** | **1）** | **2）** | **3）** | **4）** | **5）** | **6）** | **7）** | **8）** | **score** | **quality** |  |  |  |
| Geerlings, M. I. | 2001 | cohort study | ★ | ★ | ★ | ★ | ★★ | ★ | ★ | ★ | 9 | high |  |  |  |
| Senanarong, V. | 2002 | cohort study | ★ | ★ | ★ | ★ |  | ★ | ★ | ★ | 7 | high |  |  |  |
| Geerlings, M. I. | 2003 | cohort study | ★ | ★ | ★ | ★ | ★★ | ★ | ★ | ★ | 9 | high |  |  |  |
| Schupf, N. | 2003 | cohort study | ★ | ★ | ★ | ★ | ★★ | ★ |  |  | 7 | high |  |  |  |
| Rasgon, N. L. | 2005 | case-control study | ★ | ★ | ★ | ★ | ★ | ★ | ★ |  | 7 | high |  |  |  |
| Schupf, N. | 2006 | cohort study | ★ | ★ | ★ | ★ | ★★ | ★ | ★ |  | 8 | high |  |  |  |
| Ravaglia, G. | 2007 | cohort study | ★ | ★ | ★ | ★ | ★★ | ★ | ★ |  | 8 | high |  |  |  |
| Coppus, A. M. W. | 2010 | cohort study | ★ | ★ | ★ | ★ |  | ★ | ★ |  | 6 | medium |  |  |  |
| Ryan, J. | 2014 | cohort study | ★ | ★ | ★ | ★ | ★★ | ★ | ★ | ★ | 9 | high |  |  |  |
| Li, F. D. | 2016 | cohort study | ★ | ★ | ★ | ★ | ★★ | ★ | ★ | ★ | 9 | high |  |  |  |
| Paganini-Hill, A. | 2020 | cohort study |  | ★ | ★ | ★ | ★ | ★ | ★ | ★ | 7 | high |  |  |  |
| Yoo, J. E. | 2020 | cohort study | ★ | ★ | ★ | ★ | ★★ | ★ | ★ |  | 8 | high |  |  |  |
| Prince, M. J. | 2018 | cohort study | ★ | ★ | ★ | ★ | ★★ | ★ | ★ |  | 8 | high |  |  |  |
| Hong, X. | 2001 | case-control study | ★ | ★ | ★ | ★ | ★★ | ★ | ★ |  | 8 | high |  |  |  |
| Song, X. | 2020 | cohort study | ★ | ★ | ★ | ★ | ★★ | ★ | ★ | ★ | 9 | high |  |  |  |
| Shimizu, Y. | 2019 | cohort study | ★ | ★ | ★ | ★ | ★★ | ★ | ★ |  | 8 | high |  |  |  |
| Paganini-Hill, A. | 1994 | case-control study | ★ | ★ | ★ | ★ |  | ★ | ★ |  | 6 | medium |  |  |  |
| Najar, J. | 2019 | cohort study | ★ | ★ | ★ | ★ | ★★ | ★ | ★ |  | 8 | high |  |  |  |
| Gilsanz, P. | 2018 | cohort study |  | ★ | ★ | ★ | ★★ | ★ | ★ |  | 7 | high |  |  |  |
| Manly, J. J. | 2000 | case-control study | ★ | ★ | ★ | ★ | ★★ | ★ | ★ |  | 8 | high |  |  |  |

| **B) AHRQ evaluation scale for cross-sectional study** | | | | | | | | | | | | | | | |
| --- | --- | --- | --- | --- | --- | --- | --- | --- | --- | --- | --- | --- | --- | --- | --- |
| **Author** | **Publication year** | **study type** | **1）** | **2）** | **3）** | **4）** | **5)** | **6)** | **7)** | **8)** | **9)** | **10)** | **11)** | **score** | **quality** |
| Lebrun, C. E. | 2005 | cross-sectional study | ★ | ★ | ★ | ★ |  | ★ | ★ | ★ | ★ |  |  | 8 | high |
| Hu, J. | 2017 | cross-sectional study | ★ | ★ | ★ | ★ |  | ★ | ★ | ★ |  |  |  | 7 | high |
| **Notes:** In NOS, study can be given a maximum of one star for each fulfilled item within the Study selection and Outcome categories. A maximum of two stars can be assigned for Comparability. Scores range from 0 to 9. Scores < 5 were considered as low-quality studies; scores 5-6 as moderate-quality studies; scores > 7 indicated high-quality studies. In AHRQ evaluation scale, there were 11 items in total, and each item has three options: "Yes, No, Not Clear". The answer "Yes" will score 1 point, while the answer "No or Not Clear" will score 0 point. Scores range from 0 to 11. Scores < 3 were considered as low-quality studies; scores 4-7 as moderate-quality studies; scores 8-11 indicated high-quality studies. | | | | | | | | | | | | | | | |

**e Table 6. Sensitivity Analysis by the Leaving-One-Out Method**

1. **Age at menarche and all-cause dementia, Alzheimer's disease (AD) and cognitive impairment (CI)**

| **Exposure and Outcome** | **Categories** | **Excluded study** | **Results After Study Exclusion (Summary RR (95% CI))** |
| --- | --- | --- | --- |
| **Age at menarche and all-cause dementia** | **Age at menarche 13-14 vs≤12* years** |  |  |
|  |  | Rasgon, N. L,2005 | 0.95(0.84,1.07) |
|  |  | Paganini-Hil, A., 1994 | 0.93(0.83,1.03) |
|  |  | Geerlings, M. L,2001 | 0.95(0.85,1.05) |
|  |  | PaganiniHill, A. ,2020 | 0.93(0.88,0.98) |
|  |  | Yoo,J. E..2020 | 0.95(0.76,1.19) |
|  | **Age at menarche＞14vs≤12* years** |  |  |
|  |  | Rasgon, N. L,2005 | 1.04(0.82,1.31) |
|  |  | Paganini-Hil, A., 1994 | 1.02(0.96,1.08) |
|  |  | Geerlings, M. L,2001 | 1.04(0.90,1.20) |
|  |  | PaganiniHill, A. ,2020 | 1.04(0.90,1.20) |
|  |  | Yoo,J. E..2020 | 1.02(0.79,1.30) |
| **Age at menarche and AD** | **Age at menarche (13-14 vs ≤12* years)** |  |  |
|  |  | Geerlings, M. I.,2001 | 0.94(0.69,1.00) |
|  |  | Yoo,J. E.,2020 | 0.71(0.48,1.05) |
|  | **Age at menarche (≥14 vs ≤12* years)** |  |  |
|  |  | Geerlings, M. I.,2001 | 1.04(0.96,1.13) |
|  |  | Yoo,J. E.,2020 | 0.87(0.63,1.20) |
| **Age at menarche and CI** | **Age at menarche (14-15 vs <14* years)** |  |  |
|  |  | Song, X.,2020 | 1.28(0.89,1.85) |
|  |  | Shimizu, Y.,2019 | 1.12(0.97,1.29) |
|  | **Age at menarche (>15 vs <14* years)** |  |  |
|  |  | Song, X.,2020 | 1.71(0.96,3.05) |
|  |  | Shimizu, Y.,2019 | 1.17(0.92,1.49) |

1. **Age at menopause and all-cause dementia, AD and CI**

| **Exposure and Outcome** | **Categories** | **Excluded study** | **Results After Study Exclusion**  **(Summary RR (95% CI))** |
| --- | --- | --- | --- |
| **Age at menopause and all-cause dementia** | **Age at menopause (≥45 vs <45* years)** |  |  |
|  |  | Rasgon, N. L.,2005 | 0.91(0.83,0.99) |
|  |  | Paganini-Hill, A.,1994 | 0.86(0.76,0.97) |
|  |  | Coppus, A. M. W.,2010 | 0.89(0.80,0.98) |
|  |  | PaganiniHill, A.,2020 | 0.86(0.76,0.96) |
|  |  | Yoo, J. E.,2020 | 0.87(0.73,1.03) |
|  |  | Gilsanz, P.,2018 | 0.86(0.73,1.01) |
|  |  | Ryan, J.,2014 | 0.85(0.75,0.95) |
| **Age at menopause and AD** | **Age at menopause (≥45 vs <45* years)** |  |  |
|  |  | Hong, X.,2001 | 0.62(0.27,1.43) |
|  |  | Yoo, J. E.,2020 | 0.54(0.36,0.82) |
|  |  | Schupf, N.,2003 | 0.76(0.53,1.08) |
| **Age at menopause and CI** | **Age at menopause (45-49 vs <45* years)** |  |  |
|  |  | Song, X.,2020 | 0.89(0.75,1.06) |
|  |  | Shimizu, Y.,2019 | 0.80(0.68,0.94) |
|  |  | Ryan, J.,2014 | 0.80(0.61,1.05) |
|  | **Age at menopause (≥50 vs <45* years)** |  |  |
|  |  | Song, X.,2020 | 0.79(0.62,1.00) |
|  |  | Shimizu, Y.,2019 | 0.69(0.54,0.87) |
|  |  | Ryan, J.,2014 | 0.63(0.53,0.74) |

1. **Reproductive period and all-cause dementia, AD and CI**

| **Exposure and Outcome** | **Categories** | **Excluded study** | **Results After Study Exclusion (Summary RR (95% CI))** |
| --- | --- | --- | --- |
|  | **Reproductive period (≥35 vs <35* years)** |  |  |
| **Reproductive period and all-cause dementia** |  | Rasgon, N. L,2005 | 1.02(0.78,1.32) |
|  |  | Geerlings, M.I.,2001 | 0.89(0.71.1.11) |
|  |  | Yoo,J. E.,2020 | 0.97(0.65,1.45) |
|  |  | Najar, J,2019 | 0.84(0.72,0.99) |
| **Reproductive period and AD** | **Reproductive period (≥35 vs <35* years)** |  |  |
|  |  | Geerlings, M.I.,2001 | 0.96(0.69,1.34) |
|  |  | Yoo,J. E.,2020 | 1.19(0.82,1.72) |
|  |  | Najar, J.,2019 | 0.87(0.86,0.88) |
| **Reproductive period and CI** | **Reproductive period (35-39 vs <35* years)** |  |  |
|  |  | Song, X.,2020 | 0.89(0.75,1.06) |
|  |  | Shimizu, Y.,2019 | 0.80(0.68,0.94) |
|  |  | Ryan, J.,2014 | 0.80(0.61,1.05) |
|  | **Reproductive period (>39 vs <35* years)** |  |  |
|  |  | Song, X.,2020 | 0.63(0.41,0.97) |
|  |  | Shimizu, Y,2019 | 0.73(0.62,0.86) |

**e Table 7. List of studies excluded at full-text screening stage**

| Number | Author | Title | Reason |
| --- | --- | --- | --- |
| 1 | Lumley, L. A. | AGE EFFECTS ON COGNITIVE-FUNCTIONING - THE IMPORTANCE OF THE MENOPAUSE | Only Abstract |
| 2 | Halbreich, U. | POSSIBLE ACCELERATION OF AGE EFFECTS ON COGNITION FOLLOWING MENOPAUSE | Lack of information for effect values |
| 3 | Buckwalter, J. G. | Body weight, estrogen and cognitive functioning in Alzheimer's disease: An analysis of the Tacrine Study Group data | Lack of information for effect values |
| 4 | Smith, C. A. | Lifetime estrogen exposure and cognitive performance in elderly women | Lack of information for effect values |
| 5 | Yaffe, K. | Serum estrogen levels, cognitive performance, and risk of cognitive decline in older community women | Lack of information for effect values |
| 6 | Barrett-Connor, E. | Cognitive function and endogenous sex hormones in older women | Lack of information for effect values |
| 7 | Smith, C. A. | Lifelong estrogen exposure and cognitive performance in elderly women | Lack of information for effect values |
| 8 | Sobow, T. M. | Hormonal decline indicator in women (age at menopause) modifies age of onset in sporadic Alzheimer's disease | Lack of information for effect values |
| 9 | Tsai, C. K. | Female reproductive health and cognitive function | Not related to topic |
| 10 | Cunningham, C. J. | Endogenous sex hormone levels in postmenopausal women with Alzheimer's disease | Lack of information for effect values |
| 11 | Laughlin, G. A. | Endogenous oestrogens predict 4-year decline in verbal fluency in postmenopausal women: the Rancho Bernardo Study | Not related to topic |
| 12 | Patel, B. N. | Effect of menopause on cognitive performance in women with Down syndrome | Lack of information for effect values |
| 13 | Wolf, O. T. | Endogenous estradiol and testosterone levels are associated with cognitive performance in older women and men | Lack of information for effect values |
| 14 | Meyer, P. M. | A population-based longitudinal study of cognitive functioning in the menopausal transition | Lack of information for effect values |
| 15 | Hogervorst, E. | Serum levels of estradiol and testosterone and performance in different cognitive domains in healthy elderly men and women | Not related to topic |
| 16 | Patel, B. N. | Obesity enhances verbal memory in postmenopausal women with Down syndrome | Lack of information for effect values |
| 17 | Low, L. F. | Reproductive period and cognitive function in a representative sample of naturally postmenopausal women aged 60-64 years | Lack of information for effect values |
| 18 | Fuh, J. L. | A longitudinal study of cognition change during early menopausal transition in a rural community | Not related to topic |
| 19 | Kok, H. S. | Cognitive function across the life course and the menopausal transition in a British birth cohort | Not related to topic |
| 20 | Elsabagh, S. | Cognitive function in late versus early postmenopausal stage | Not related to topic |
| 21 | Luetters, C. | Menopause transition stage and endogenous estradiol and follicle-stimulating hormone levels are not related to cognitive performance: Cross-sectional results from the study of women's health across the nation (SWAN) | Lack of information for effect values |
| 22 | Marinho, R. M. | Effects of estradiol on the cognitive function of postmenopausal women | Lack of information for effect values |
| 23 | Heys, M. | Life long endogenous estrogen exposure and later adulthood cognitive function in a population of naturally postmenopausal women from Southern China: the Guangzhou Biobank Cohort Study | Not related to topic |
| 24 | Ryan, J. | Executive functions in recently postmenopausal women: Absence of strong association with serum gonadal steroids | Not related to topic |
| 25 | Hesson, J. | Cumulative estrogen exposure and prospective memory in older women | Not related to topic |
| 26 | Ryan, J. | Hormone levels and cognitive function in postmenopausal midlife women | Not related to topic |
| 27 | Tuomisto, H. | The association of serum oestradiol level, age, and education with cognitive performance in peri- and late postmenopausal women | Not related to topic |
| 28 | Dang, H. | Reproductive history correlates with late-life cognitive function in postmenopausal women | Only Abstract |
| 29 | Fox, M. | Cumulative estrogen exposure, number of menstrual cycles, and Alzheimer's risk in a cohort of British women | Lack of information for effect values |
| 30 | Rubin, L. H. | Investigation of menopausal stage and symptoms on cognition in human immunodeficiency virus-infected women | Not related to topic |
| 31 | Bojar, I. | Correlation between intensity of menopausal symptoms and cognitive domain assessed with CNS-VS tests | Not related to topic |
| 32 | Unkenstein, A. E. | Understanding women's experience of memory over the menopausal transition: subjective and objective memory in pre-, peri-, and postmenopausal women | Not related to topic |
| 33 | Rachmiyani, I. | Later age at menopause was associated with higher cognitive function in post-menopausal women | Lack of information for effect values |
| 34 | Gorelik, A. | MENOPAUSE AND COGNITION | Only Abstract |
| 35 | Ilango, S. D. | Pregnancy history and cognitive aging among older women: the Rancho Bernardo Study | Lack of information for effect values |
| 36 | Matyi, J. M. | Lifetime estrogen exposure and cognition in late life: the Cache County Study | Lack of information for effect values |
| 37 | Arsani, Nlka | Impaired cognitive function and its relationship with menopausal onset and exercise intensity of elderly women | Not related to topic |
| 38 | Jaff, N. G. | Menopausal symptoms, menopausal stage and cognitive functioning in black urban African women | Lack of information for effect values |
| 39 | Sujarwoto, S. | Premature natural menopause and cognitive function among older women in Indonesia | Lack of information for effect values |
| 40 | Henderson, V. W. | Cognition, mood, and physiological concentrations of sex hormones in the early and late postmenopause | Not related to topic |
| 41 | Hestiantoro, Andon | Low estradiol levels escalate menopausal symptoms leading to mild cognitive impairment in postmenopausal women | Lack of information for effect values |
| 42 | Yoo, J. E. | Female reproductive factors and the risk of dementia: a nationwide cohort study | Duplicate publication |

**e Table 8. Meta-analysis of Observational Studies in Epidemiology (MOOSE) checklist.**

| **Criteria** | | **Brief description of how the criteria were handled in the meta-analysis** |
| --- | --- | --- |
| **Reporting of background should include** | |  |
| 1 | Problem definition | Sex differences have been shown in the epidemiology of dementia, endogenous estrogen exposure might contribute to the elevated risk of dementia for women. Associations between endogenous estrogen indicators and risk of subtypes of dementia have been unclear. |
| 2 | Hypothesis statement | Timing of menarche and menopause, length of reproductive period and concentration of endogenous estrogen after menopause might be associated with all-cause dementia and cognitive impairment in postmenopausal women. |
| 3 | Description of study outcomes | All-cause dementia, Alzheimer's disease (AD), vascular dementia (VD) and cognitive impairment (CI). |
| 4 | Type of exposure or intervention used | Age at menarche, Age at menopause, Reproductive period, postmenopausal estradiol level. |
| 5 | Type of study designs used | Case-control/ cross-sectional / longitudinal cohort study. |
| 6 | Study population | Women in middle or later life with natural menopause. |
| **Reporting of search strategy should include** | |  |
| 7 | Qualifications of searchers (eg, librarians and investigators) | The credentials and affiliations of the investigators are provided in the author list. |
| 8 | Search strategy, including time period included in the synthesis and keywords | PubMed, Embase, and the Web of Science Core Collection were searched using a combination of search terms as following up to 12th November 2021. |
| 9 | Effort to include all available studies, including contact with authors | We contacted authors for providing additional data which we needed but not presented in article for analysis. If the required data (e.g. cases, person) for the meta-analysis were not readily available in the published article, crude numbers were calculated by means of 2x2 tables and effect sizes presented in the articles. |
| 10 | Databases and registries searched | PubMed, Embase, and the Web of Science Core Collection. |
| 11 | Search software used, name and version, including special features used (eg, explosion) | No special search software was used. EndNote was used to merge retrieved citations and eliminate duplications。 |
| 12 | Use of hand searching (eg, reference lists of obtained articles) | Hand searching was used to screen 65 full texts. |
| 13 | List of citations located and those excluded, including justification | Details of the literature search process and excluded studies are outlined in the flow chart (Figure 1). |
| 14 | Method of addressing articles published in languages other than English | We search only in the English database. |
| 15 | Method of handling abstracts and unpublished studies | The relevant literature searching was not limited to full-text literature. Conference abstracts that meet the inclusion criteria were also further screened. If necessary information lacked, we would contact author via e-mail. |
| 16 | Description of any contact with authors | Authors were contacted for providing additional data which we needed but not presented in article for analysis.  The e-mail address of the corresponding author or co-authors was used. |
| **Reporting of methods should include** | |  |
| 17 | Description of relevance or appropriateness of studies assembled for assessing the hypothesis to be tested | Observational studies were selected when exposure variable was at least one of age at menarche, age at menopause, reproductive duration, or concentration of endogenous estrogen after menopause; the endpoint of interest was all-cause dementia, AD, VD, or CI. At the same time, the study must reported an effect size (e.g., odds ratio (OR), relative risk (RR) or hazard ratio (HR)) and the corresponding 95% CI for the association between exposure and endpoint variable. |
| 18 | Rationale for the selection and coding of data (eg, sound clinical principles or convenience) | Data extracted from each of the studies were relevant to the population characteristics, study design, exposure, outcome, covariates adjusted, estimates (adjusted OR, RR, HR) and their 95% CIs, cases, controls, total number of people. |
| 19 | Documentation of how data were classified and coded (eg, multiple raters, blinding, and inrerrater reliability) | Data were extracted and analyzed by independent reviewers. They worked with blinding. Final decision was reached by consensus. |
| 20 | Assessment of confounding (eg, comparability of cases and controls in studies where appropriate) | The adjustment factors for each study were provided in descriptive eTables 1-3. Subgroup analysis was also conducted on potential confounding factors such as study type, race. Conducted sensitivity analyses by Leaving-One-Out Method, eliminating studies that had medium quality, and Mantel-Haenszel Weighting. |
| 21 | Assessment of study quality, including blinding of quality assessors; stratification or regression on possible predictors of study results | Two reviewers worked with blinding. Study quality was assessed through the Newcastle-Ottawa scale and the Agency for Healthcare Research and Quality (AHRQ) recommended scale. Results of study quality are analytically presented in eTable 5. Subgroup analysis was conducted on potential confounding factors such as study type, race, age. (eFigures 2-4) |
| 22 | Assessment of heterogeneity | Heterogeneity of the studies was assessed using Cochran Q of heterogeneity and I^2^ statistic. |
| 23 | Description of statistical methods (eg, complete description of fixed or random effects models, justification of whether the chosen models account for predictors of study results, dose-response models, or cumulative meta-analysis) in sufficient detail to be replicated | Description of methods of meta-analyses, dose-response meta-analysis, subgroup and sensitivity analyses and assessment of publication bias are detailed in the “Statistical analysis” section. |
| 24 | Provision of appropriate tables and graphics | Five figures were presented in the main manuscript; other results were provided as supplementary document. |
| **Reporting of results should include** | |  |
| 25 | Graph summarizing individual study estimates and overall estimate | Figure 1 |
| 26 | Table giving descriptive information for each study included | Table 1; eTables 1-3 |
| 27 | Results of sensitivity testing (eg, subgroup analysis) | eTable 6; eFigures 5,6 |
| 28 | Indication of statistical uncertainty of findings | 95% confidence intervals were presented with all summary effect estimates, I^2^ values and results of sensitivity analyses. |
| **Reporting of discussion should include** | |  |
| 29 | Quantitative assessment of bias (eg, publication bias) | Results, “Publication bias” section and eFigure 7. |
| 30 | Justification for exclusion (eg, exclusion of non-English-language citations) | We excluded articles with animal experiments or clinical experiments; using ERT/HET during investigates; unable to provide sufficient sample information. |
| 31 | Assessment of quality of included studies | The shortcomings of the individual studies reflected upon their quality ratings are discussed in a detailed paragraph (eTable 5) |
| **Reporting of conclusions should include** | |  |
| 32 | Consideration of alternative explanations for observed results | Study type, race may act as potential confounders. |
| 33 | Generalization of the conclusions (ie, appropriate for the data presented and within the domain of the literature review) | Most of the studies come from the USA and Europe, and evidence from other regions still need to be accumulated |
| 34 | Guidelines for future research | More investigates in large population are needed to examine the association of endogenous estrogen exposure indicators with dementia subtypes. Estrogen exposure indicators before or after menopause might have different effect on VD, with former protective and latter non or harmful. |
| 35 | Disclosure of funding source | Start-up Foundation for Scientific Research in Shandong University. |

**e Figure 1. Dose-response meta-analyses for the relationship between reproductive factors and Alzheimer's disease (AD) and cognitive impairment (CI)**


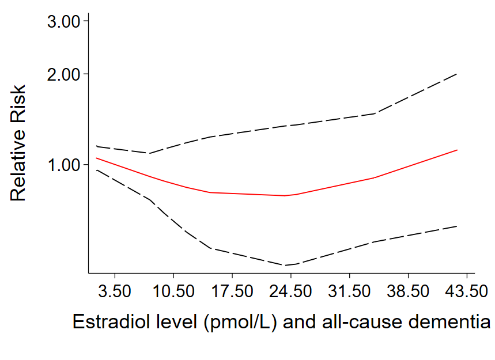


**B**

A

**I**

**H**

**G**

**F**

**E**

**D**

**C**

e Figure 2. Subgroup Analysis by Design

**(1) Age at menarche (>12 vs ≤12* years) and all-cause dementia, AD and CI**

A) Case-control studies


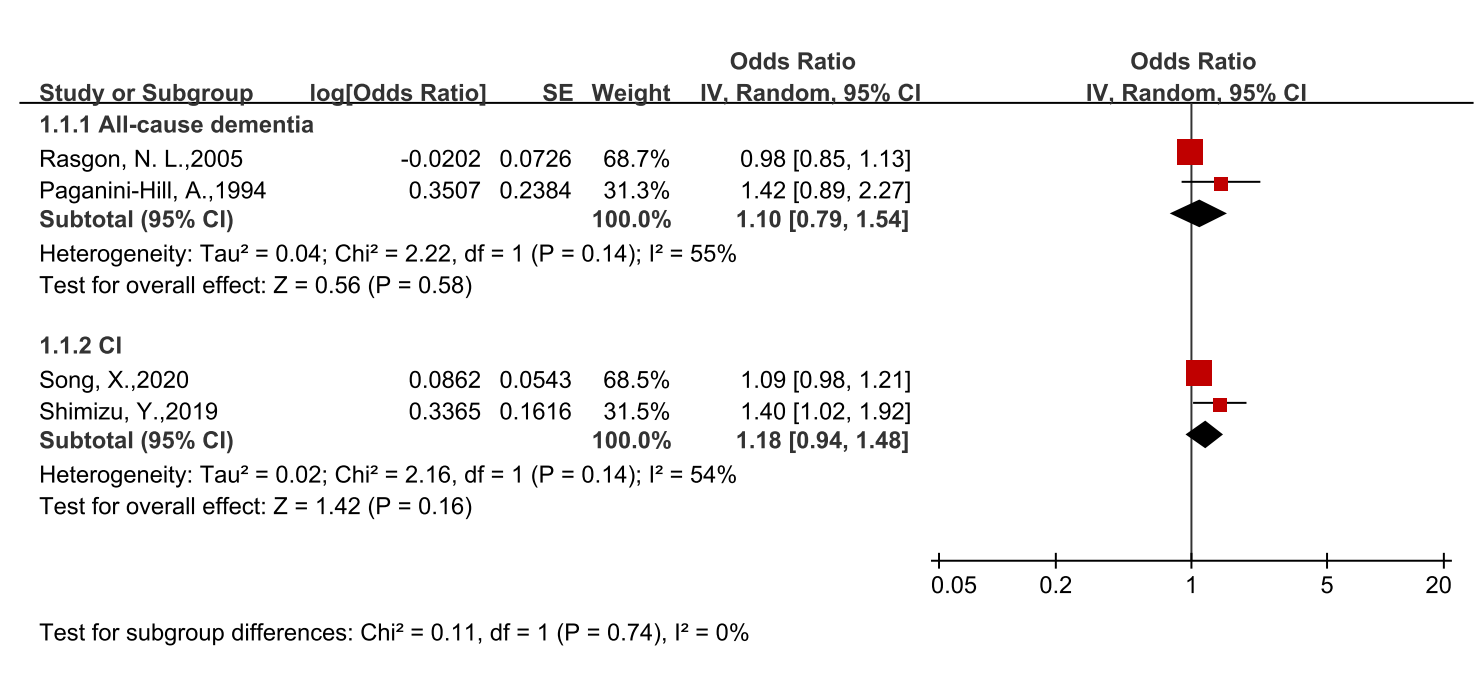


B) Cohort studies


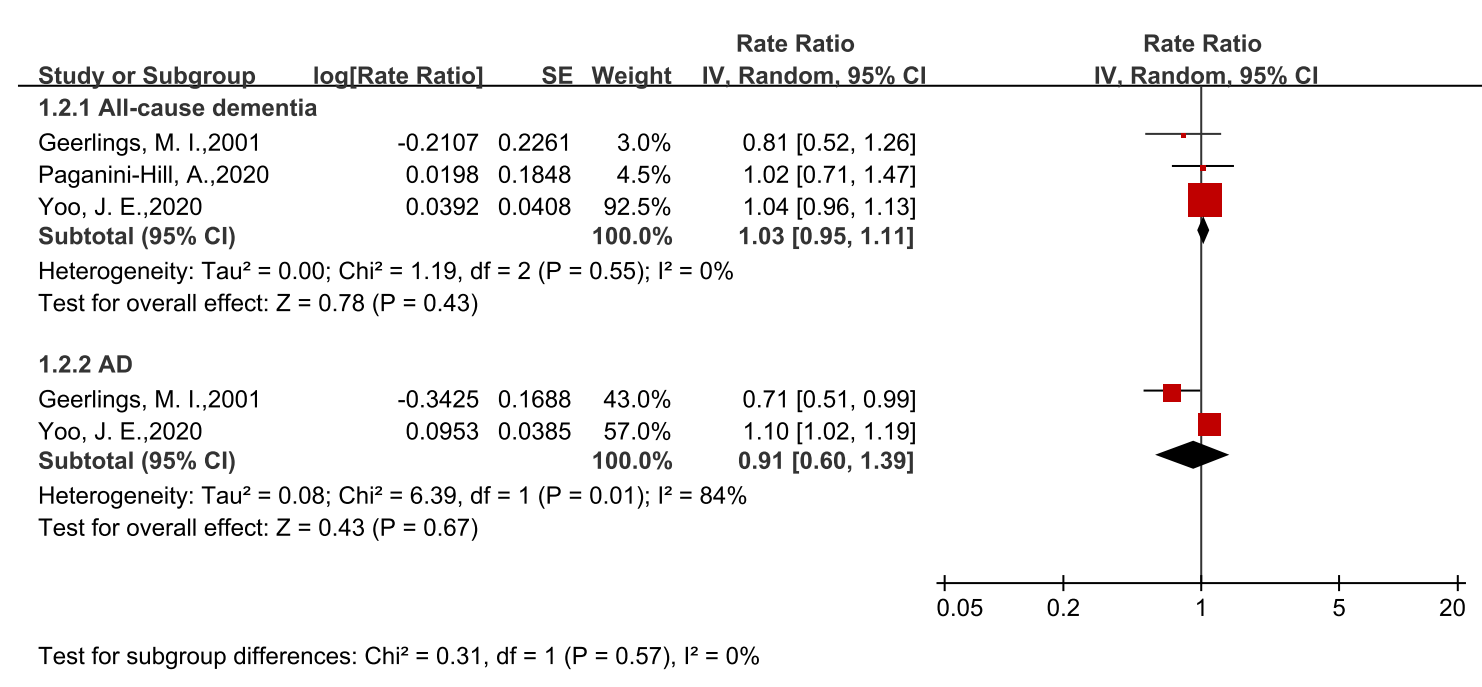


1. **Age at menopause (≥45 vs＜45* years) and all-cause dementia, AD and CI**

A) Case-control studies


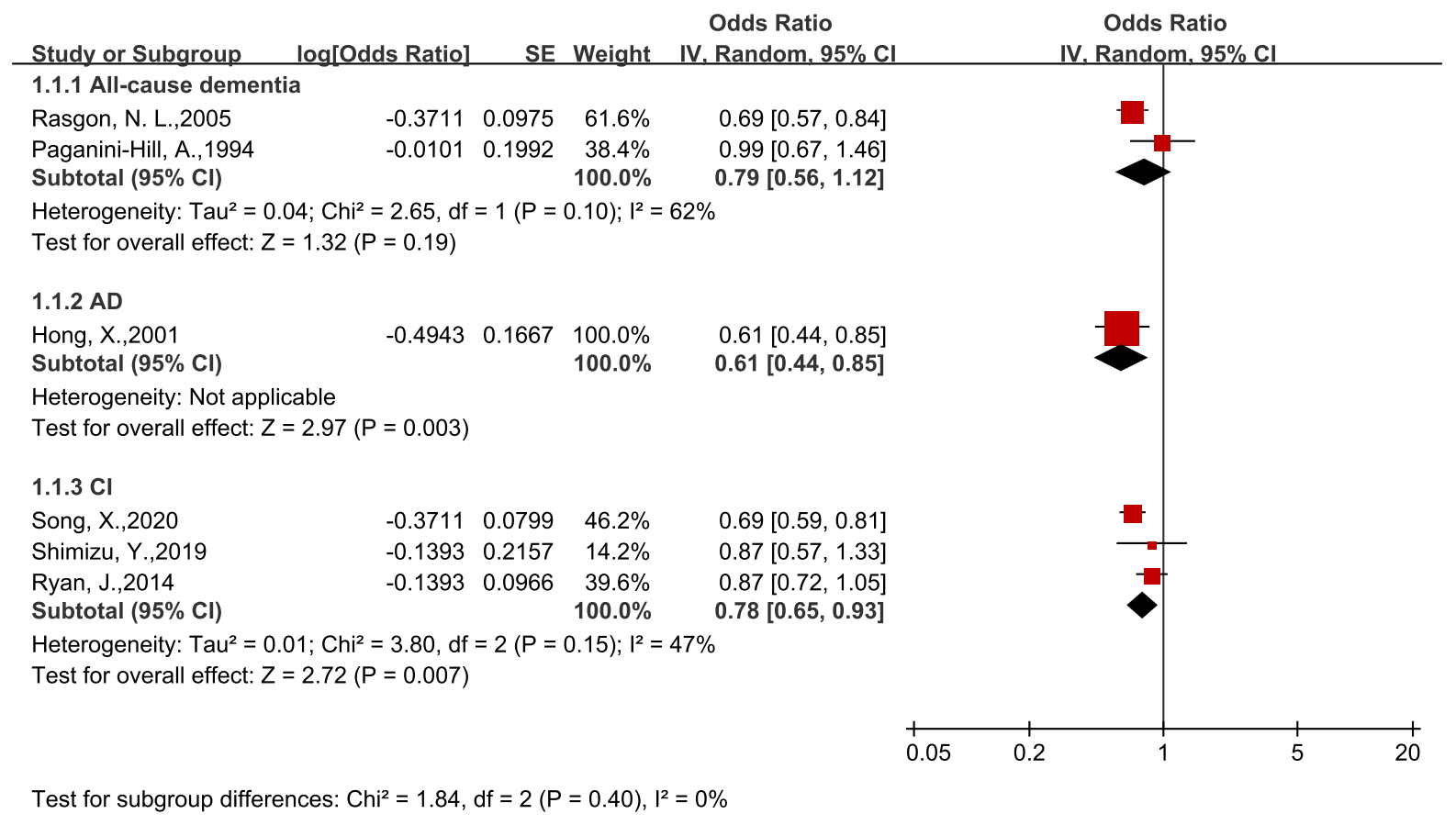


B) Cohort studies


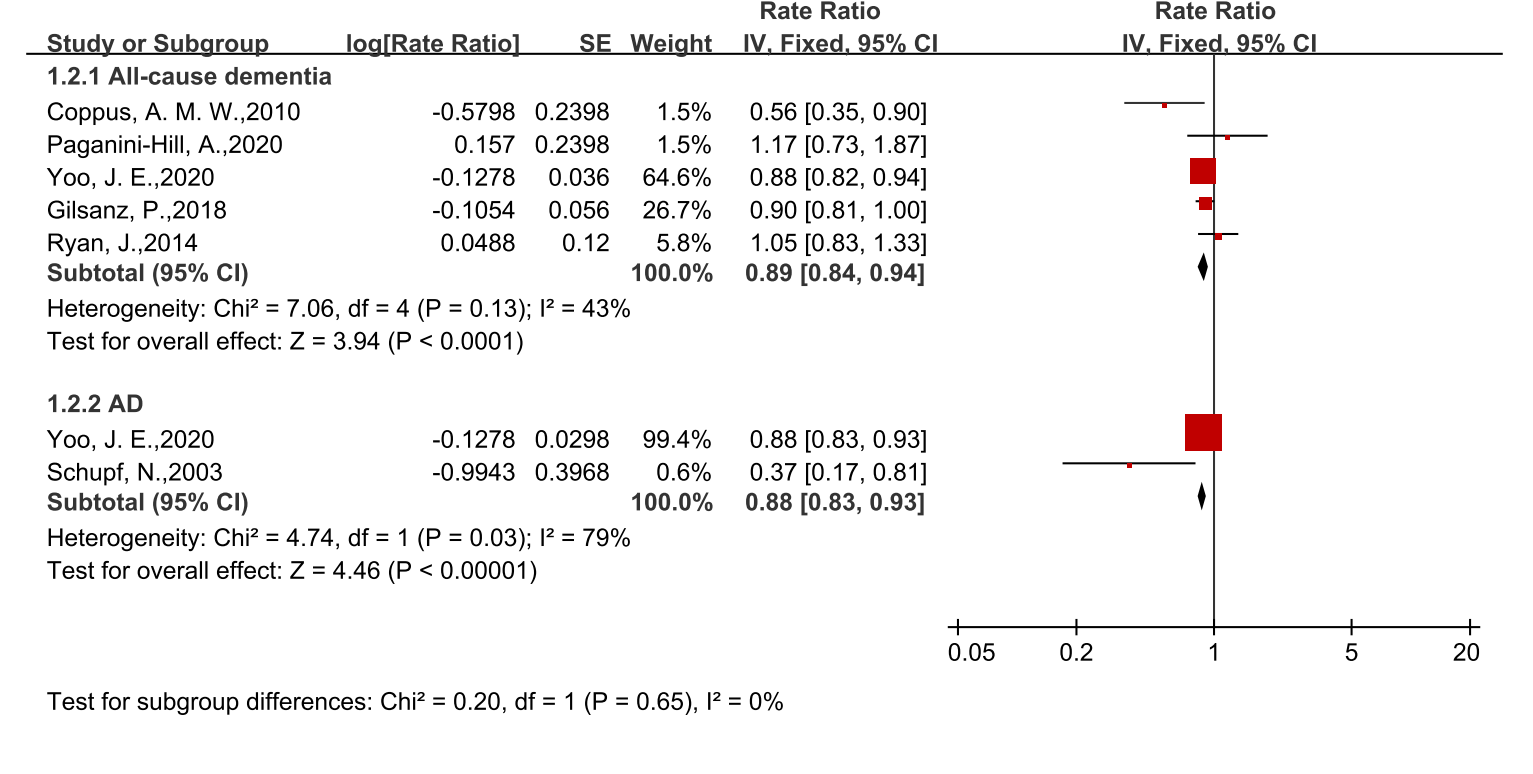


**(3) Reproductive period (≥35 vs＜35* years) and all-cause dementia, AD and CI**

A) Case-control studies


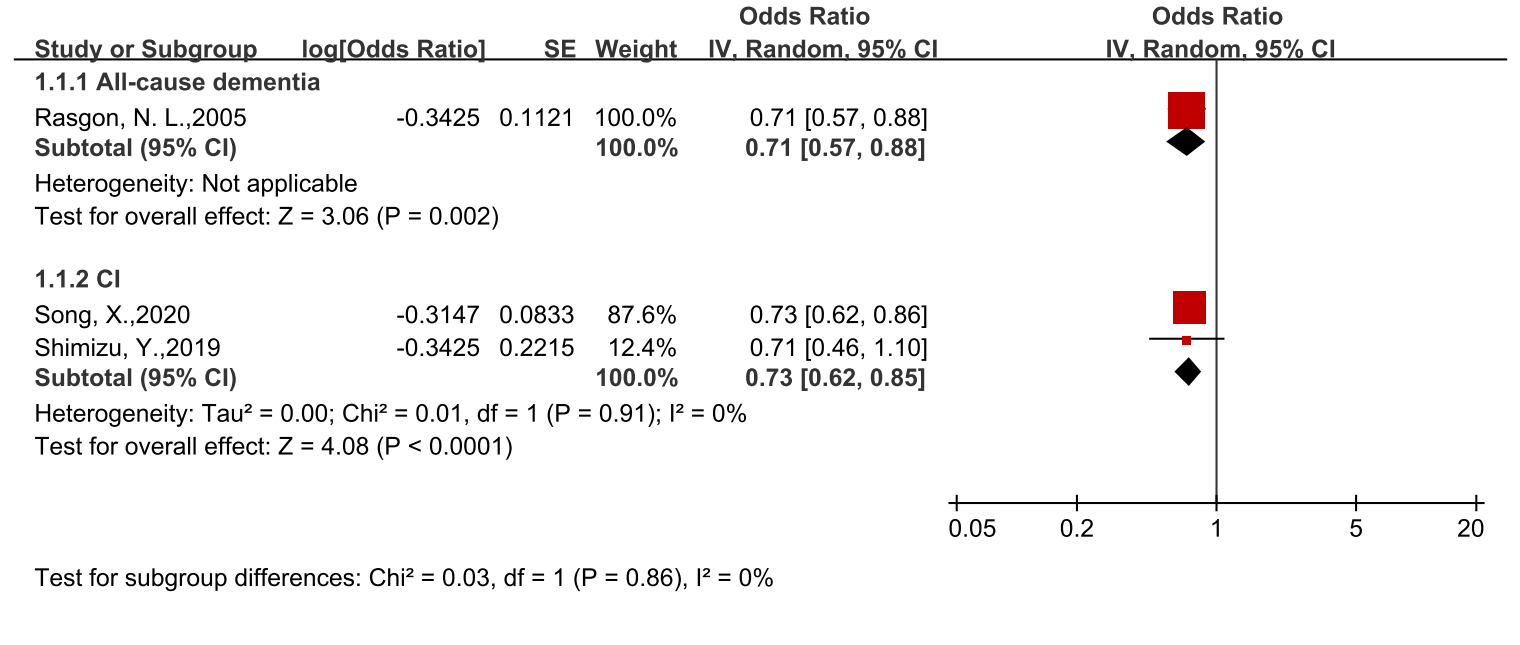


B) Cohort studies


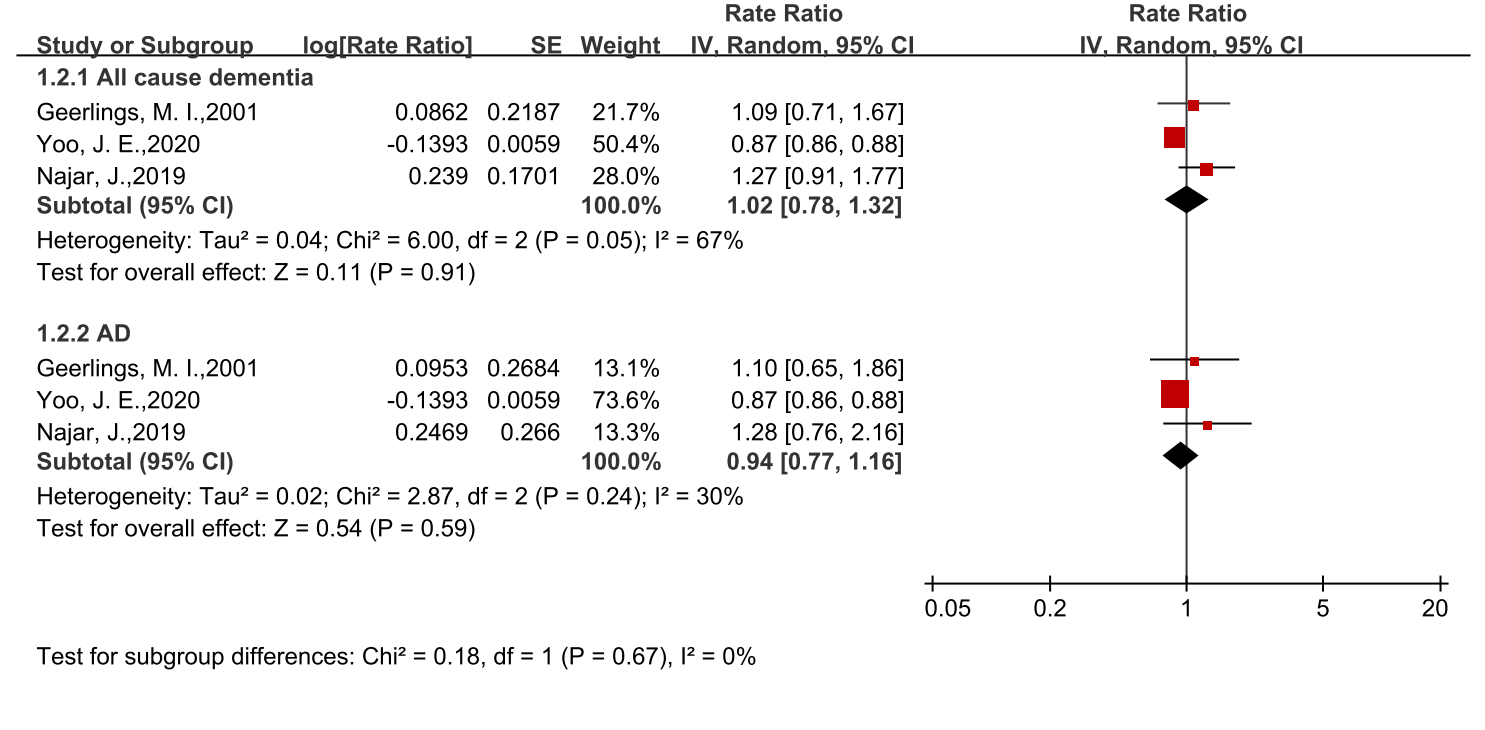


e Figure 3. Subgroup Analysis by Race

1. **Age at menarche (>12 vs ≤12* years) and all-cause dementia, AD and CI**
2. White
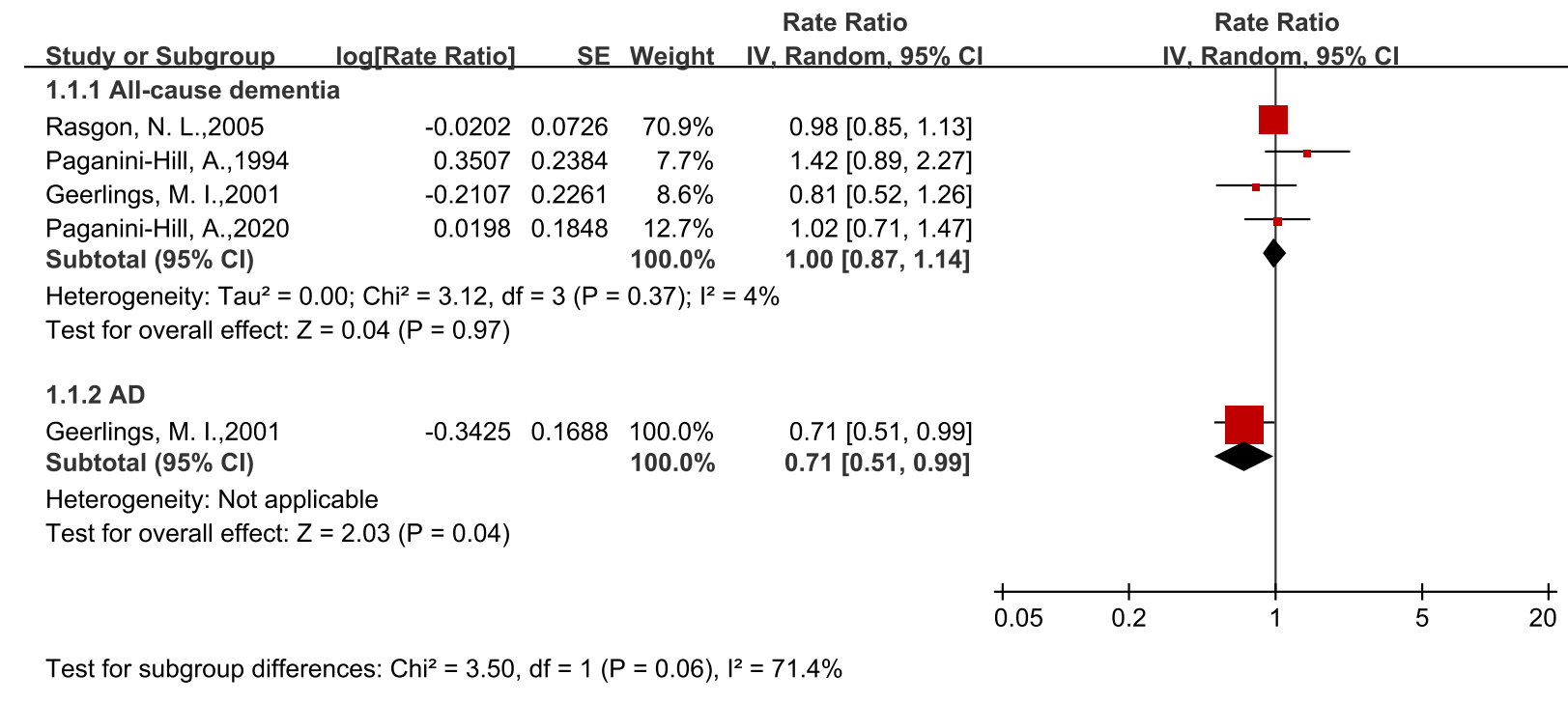


CI ：Age at menarche (>14 vs ≤14* years)

1. Nonwhite


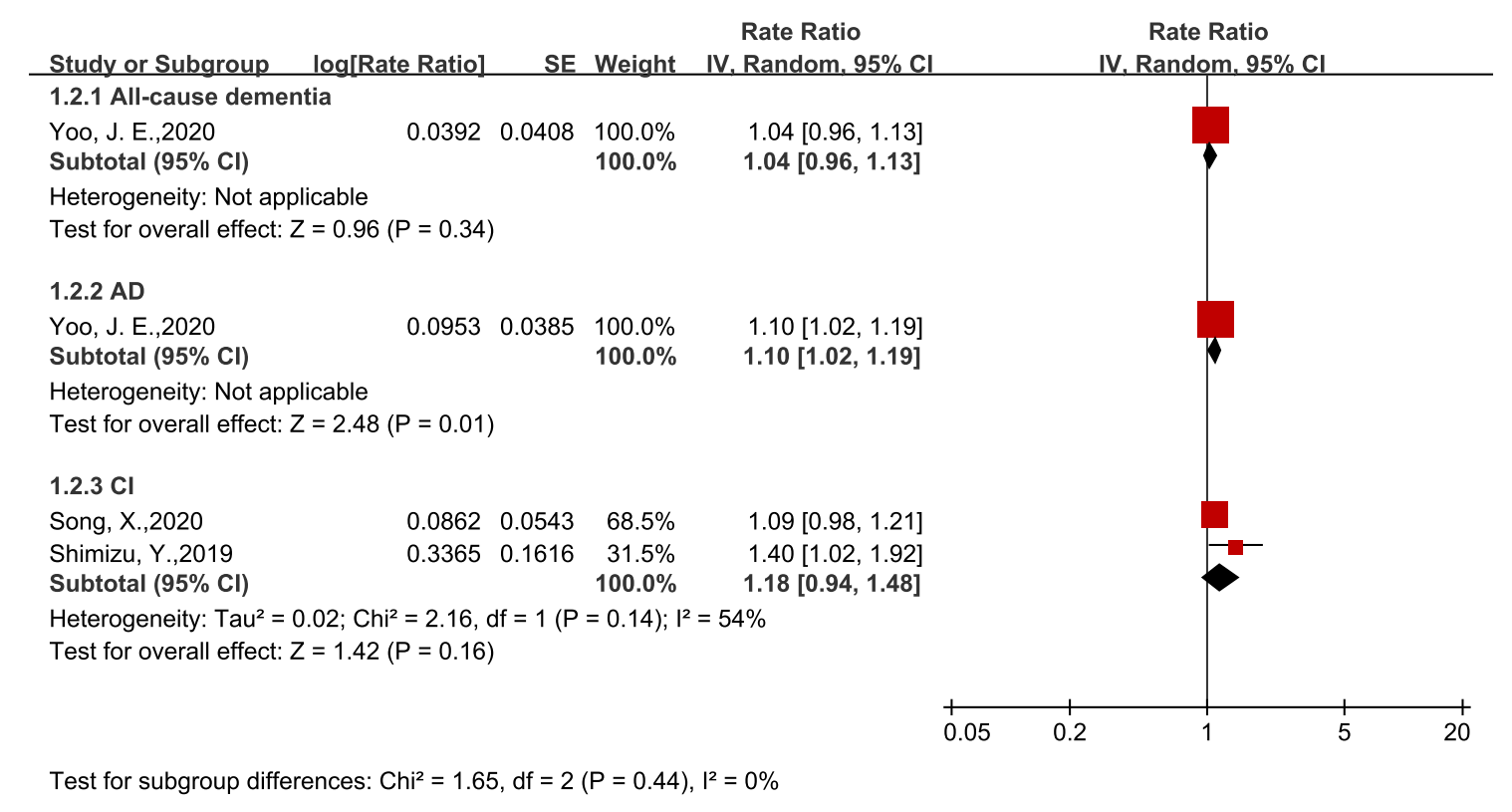


1. **Age at menopause (****≥45 vs＜45* years) and all-cause dementia, AD and CI**
2. White

^
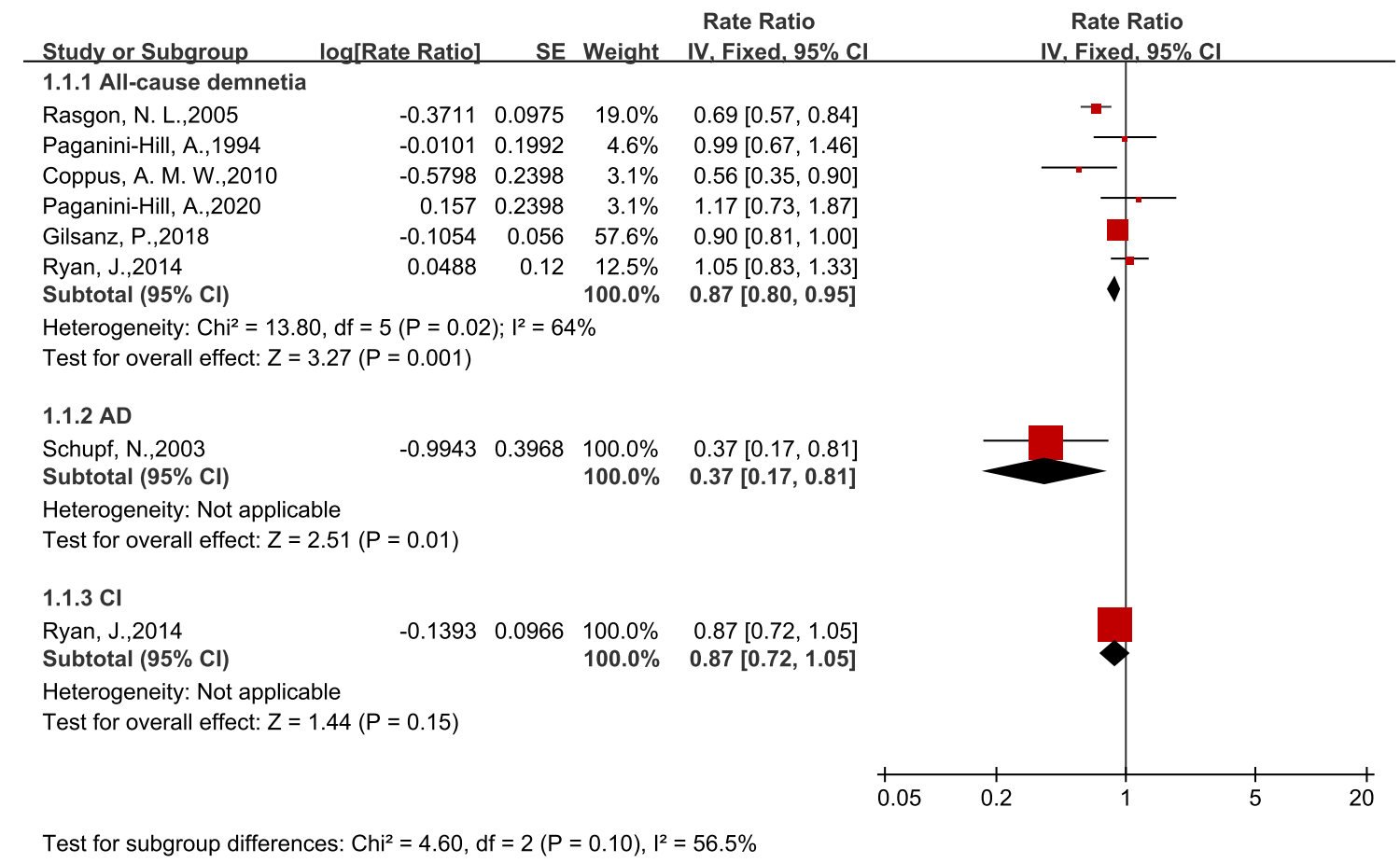
^

1. Nonwhite


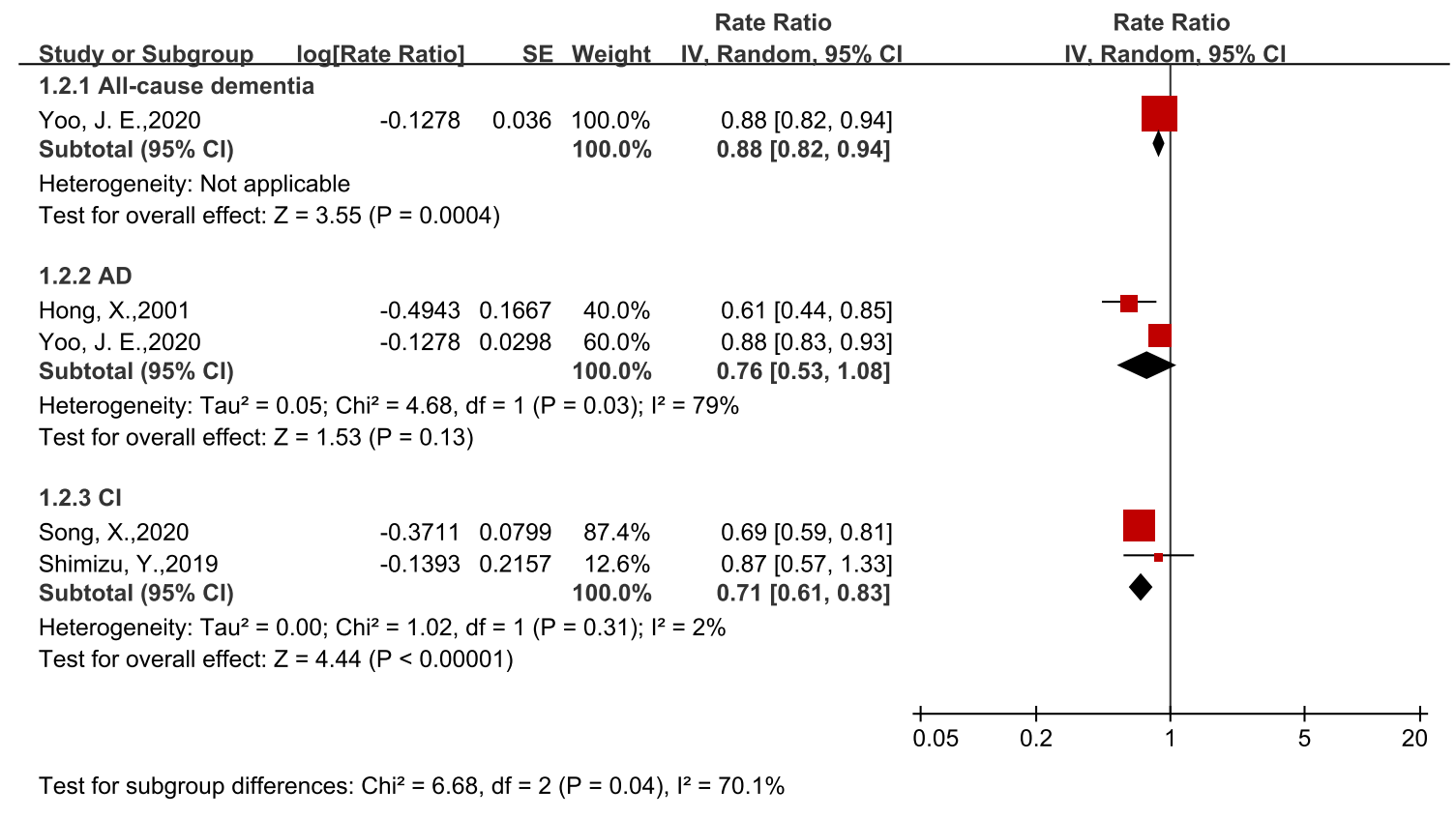


**(3) Reproductive period (≥35 vs＜35* years) and all-cause dementia, AD and CI**

A) White


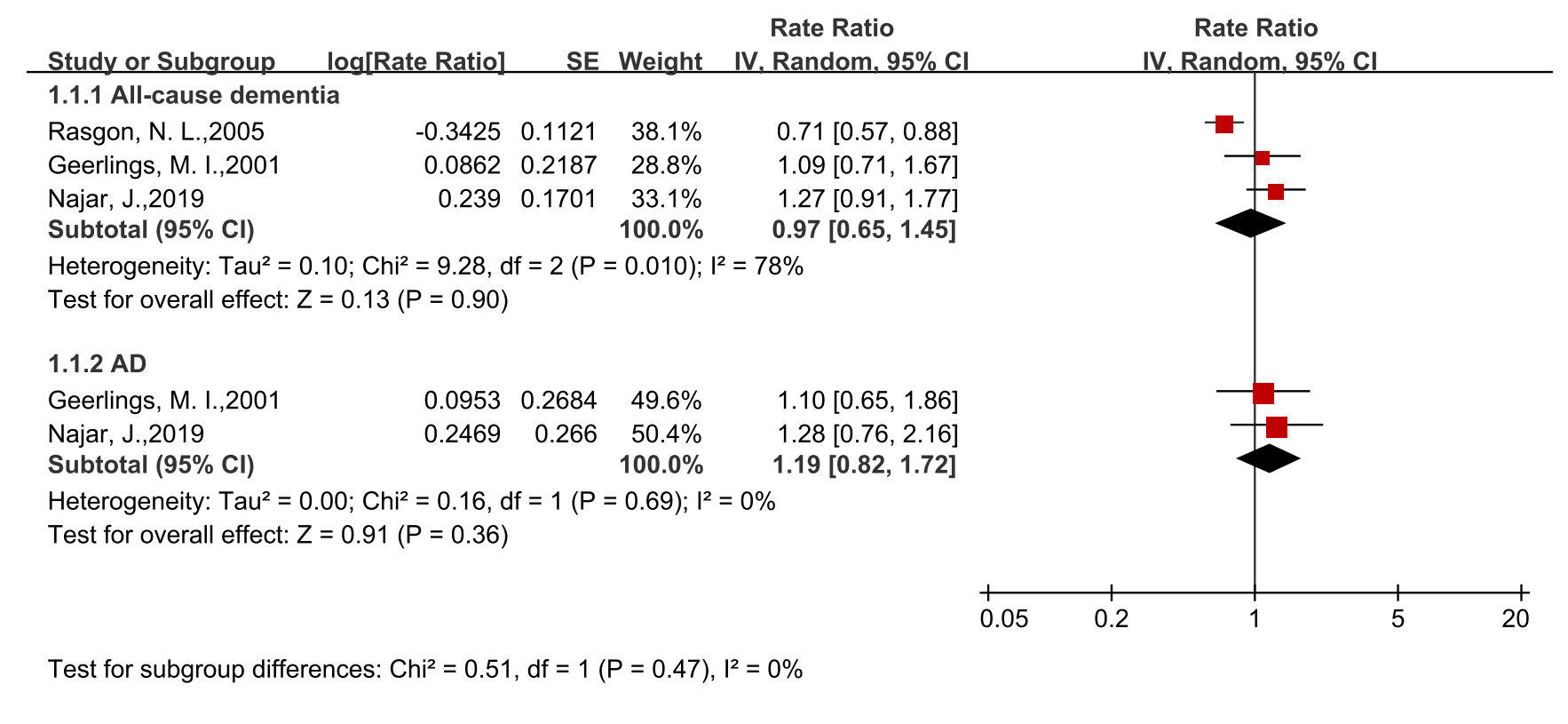


1. Nonwhite


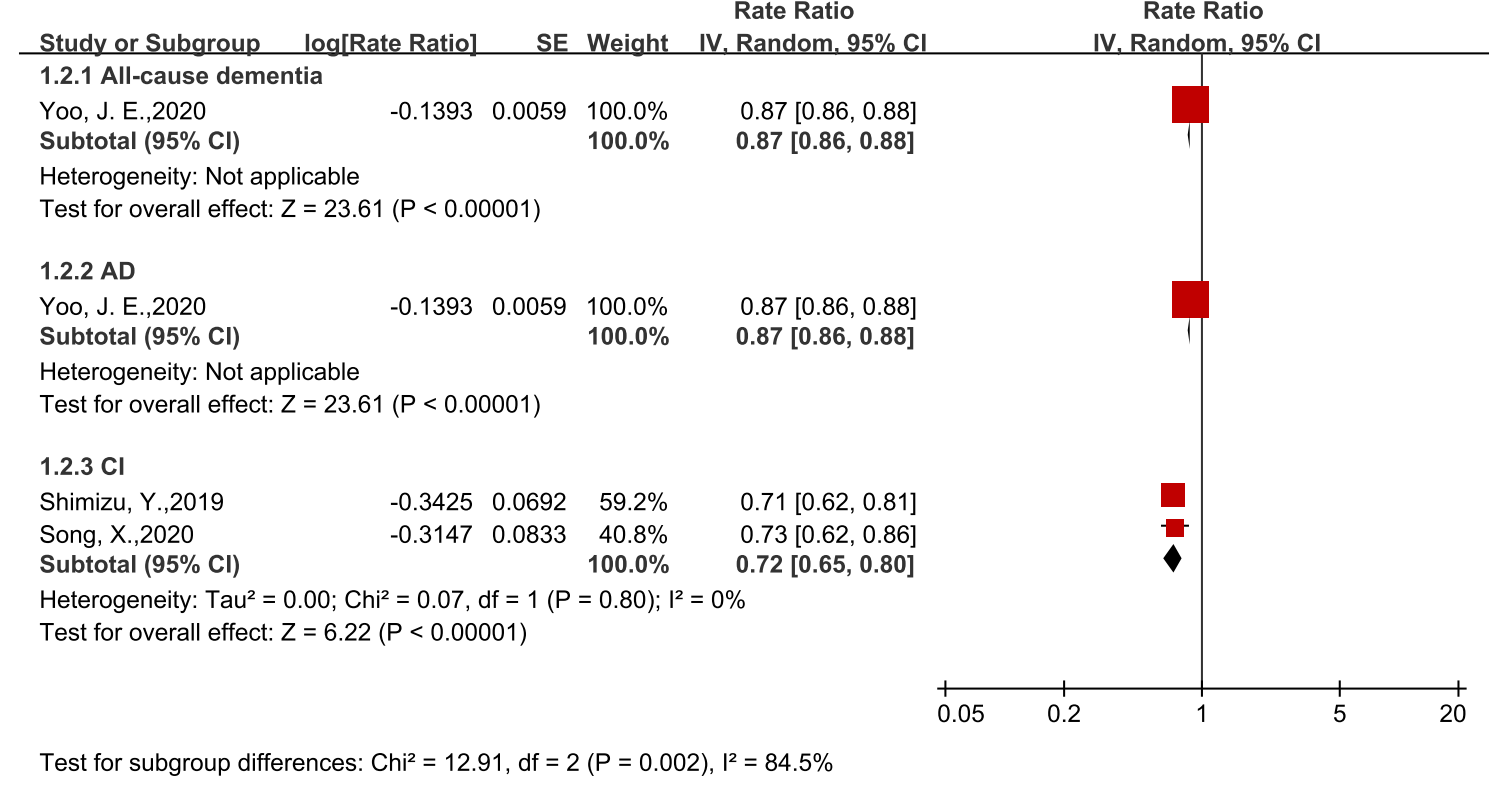


**e Figure 4. Subgroup Analysis by Age**

**(1) Age at menarche (>12 vs ≤12* years) and all-cause dementia, AD and CI**


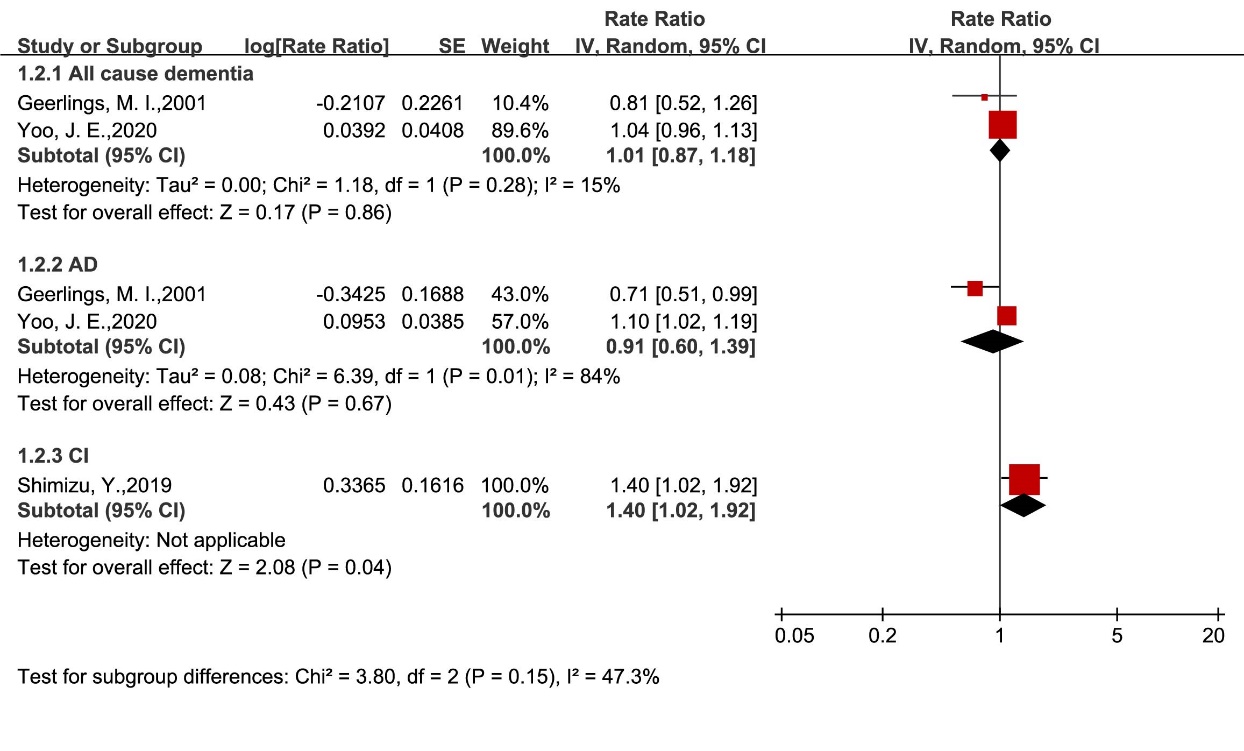


A) Age <70 years

B) Age ≥70 years


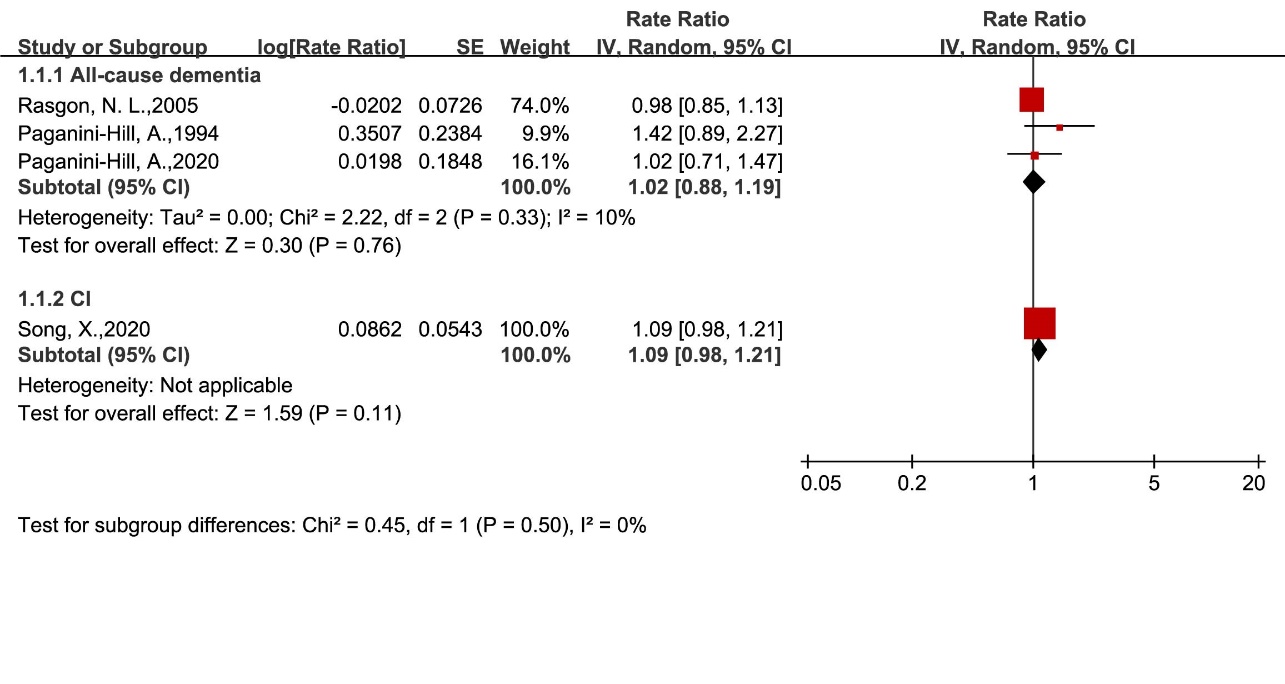


**(2) Age at menopause (≥45 vs＜45* years) and all-cause dementia, AD and CI**


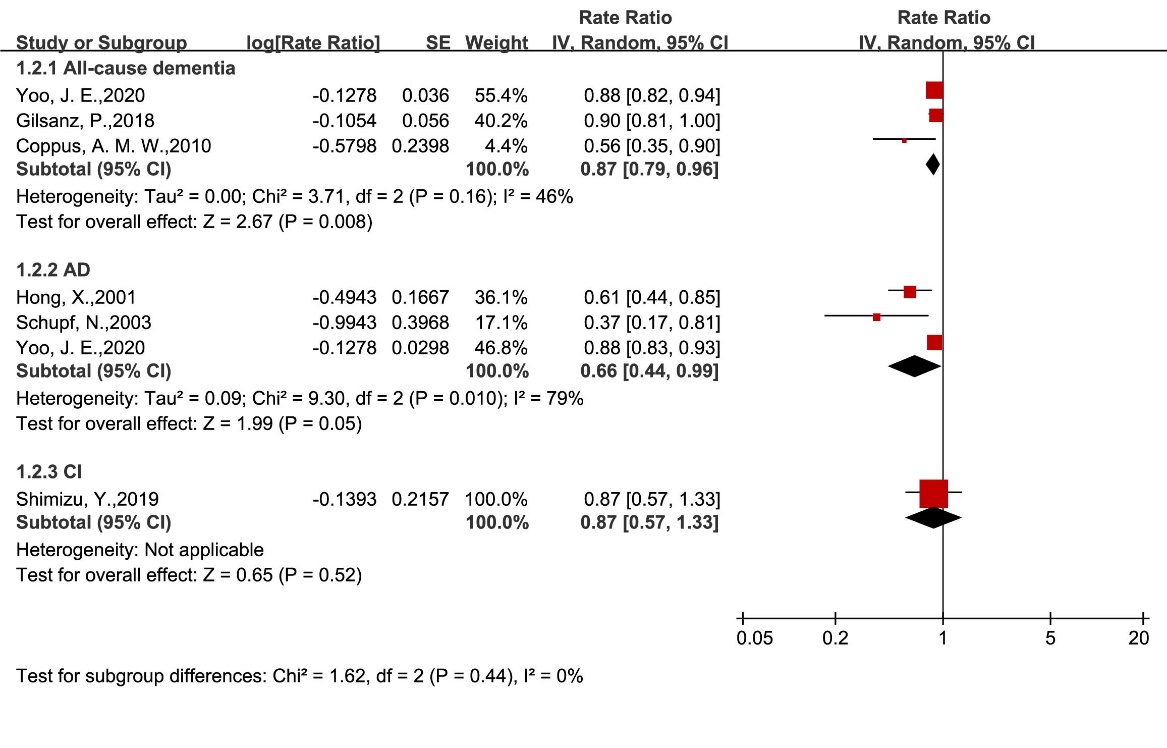


A) Age <70 years

B) Age ≥70 years


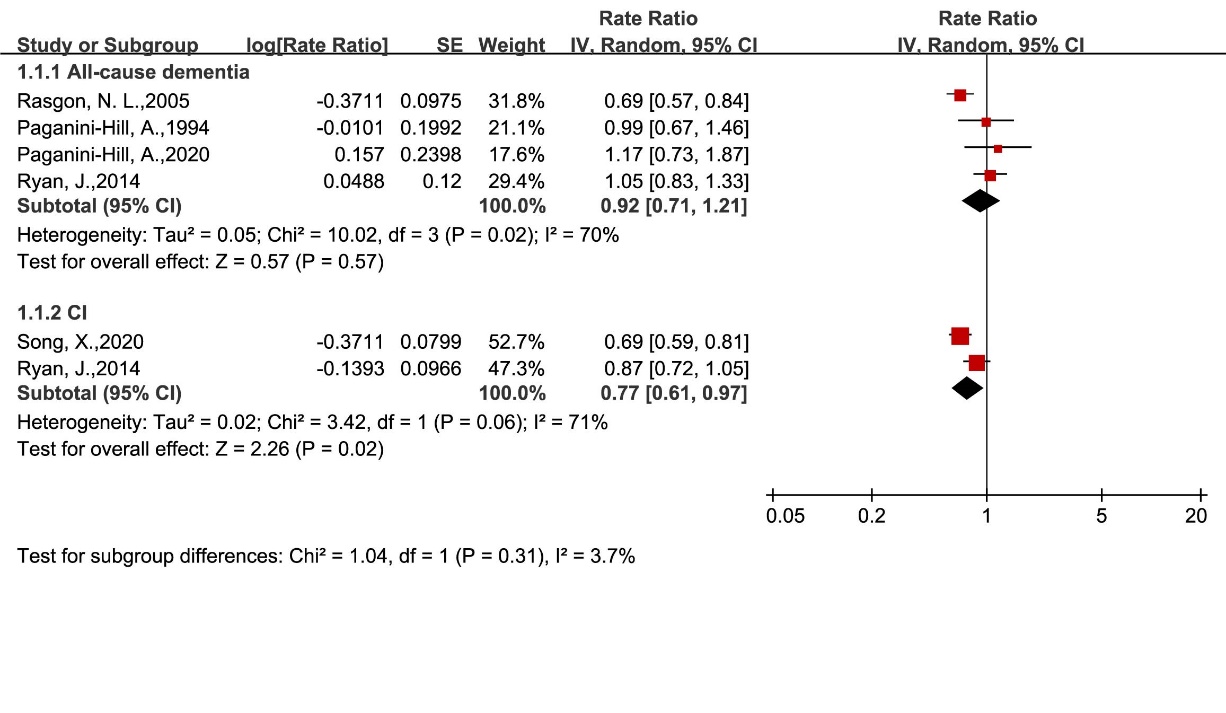


**(3) Reproductive period (≥35 vs＜35* years) and all-cause dementia, AD and C**


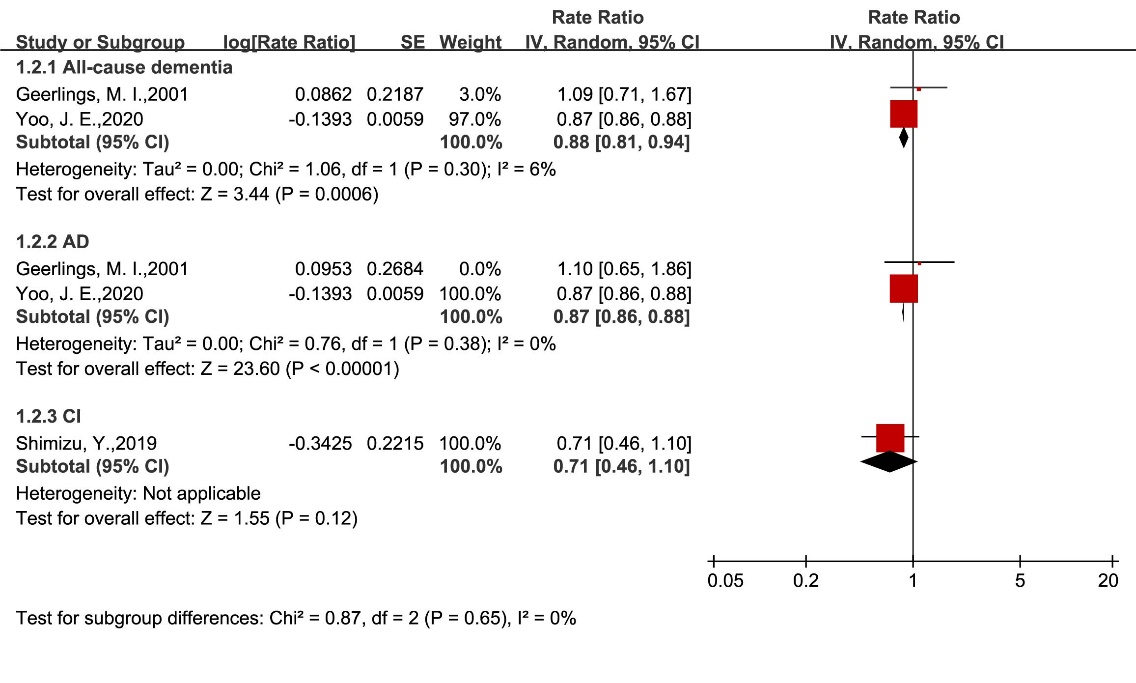


A) Age <70 years

B) Age ≥70 years


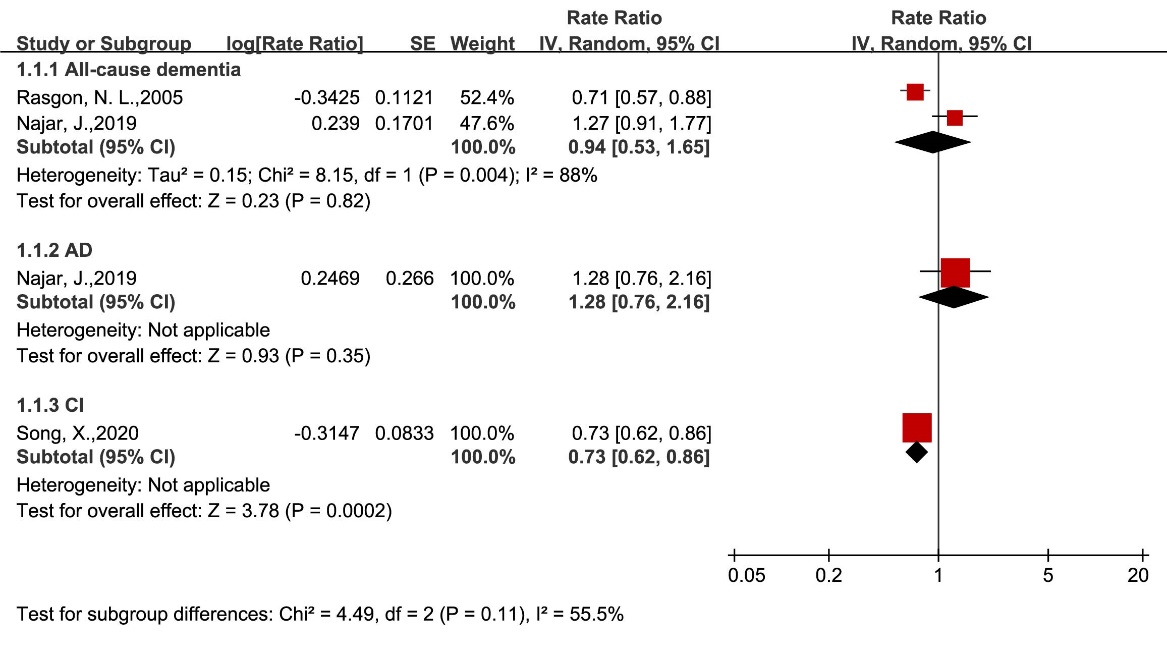


**e Figure 5. Sensitivity Analysis of Studies With High Quality**

**(1) Age at menarche and all-cause dementia, AD and CI**


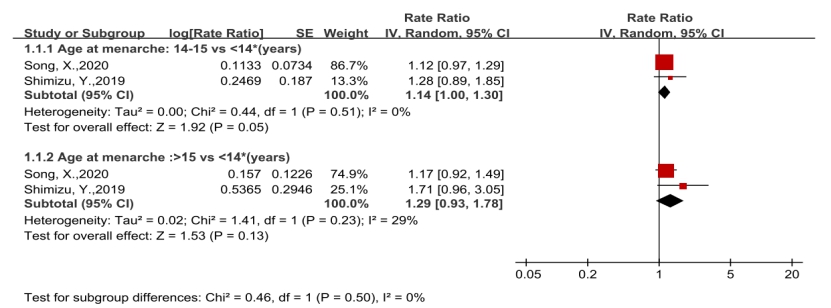


C) CI


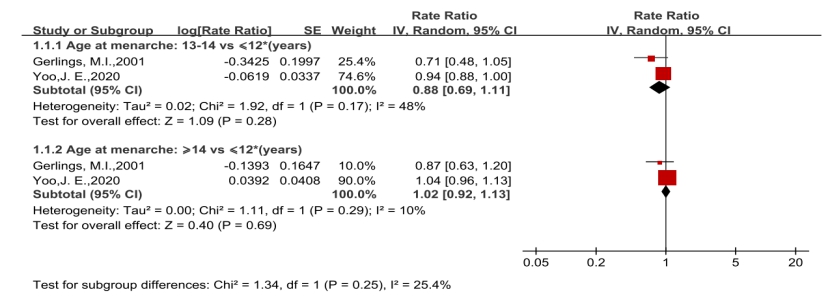


B) AD

A) All-cause dementia


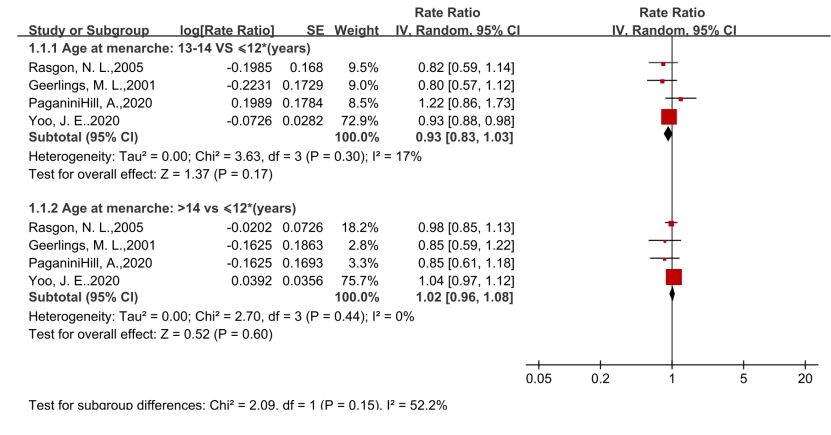


**(2) Age at menopause (≥45 vs <45* years) and all-cause dementia, AD and CI**


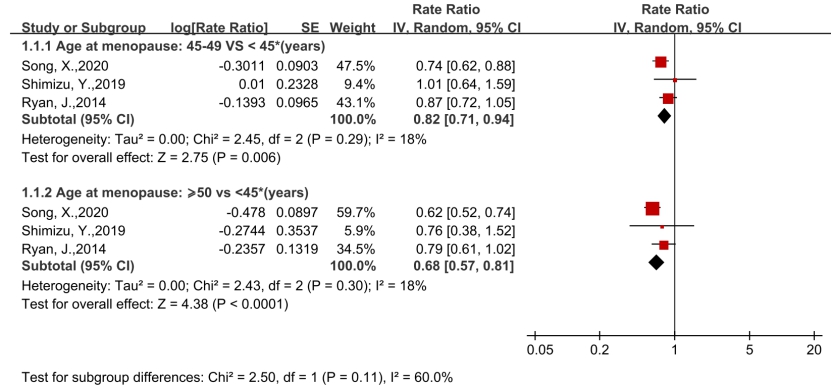


C) CI


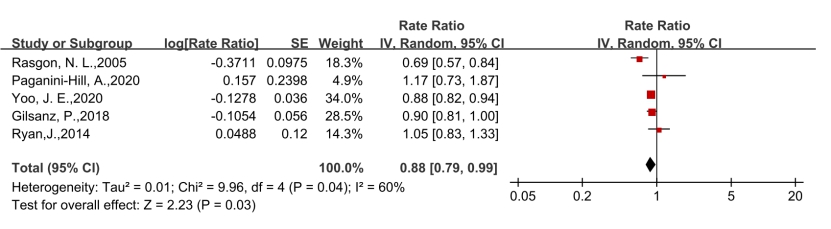


A) All-cause dementia


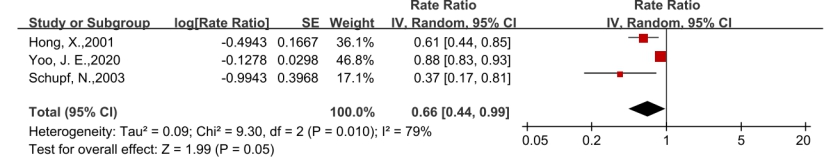


B) AD

**(3) Reproductive period (≥35 vs <35* years) and all-cause dementia, AD and CI**


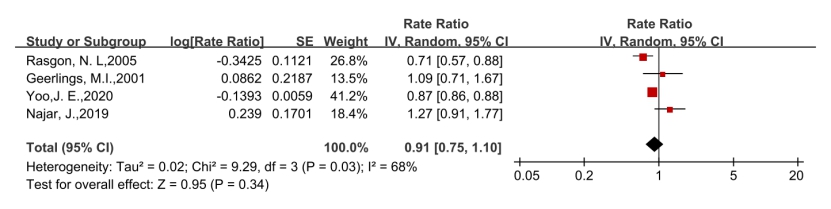
A) All-cause dementia


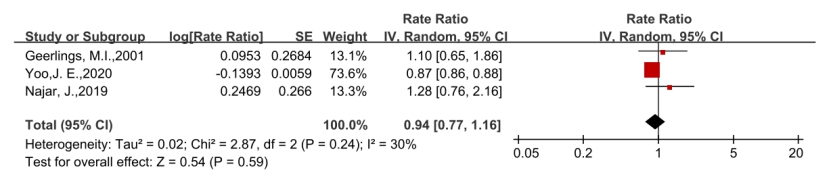
B) AD

C) CI


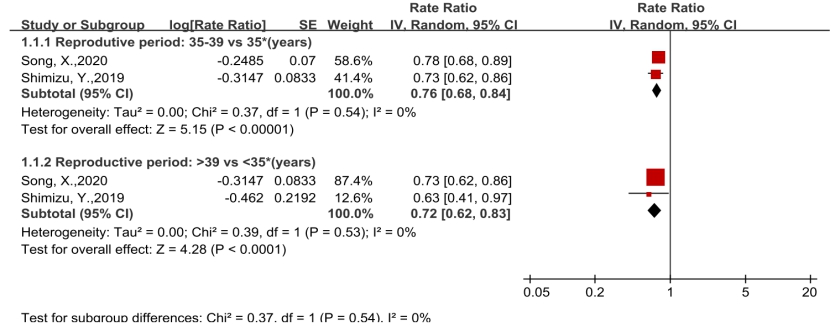


**e Figure 6. Sensitivity Analysis by Mantel-Haenszel Weighting**

(1) **Age at menarche and all-cause dementia, AD and CI**

A) All-cause dementia


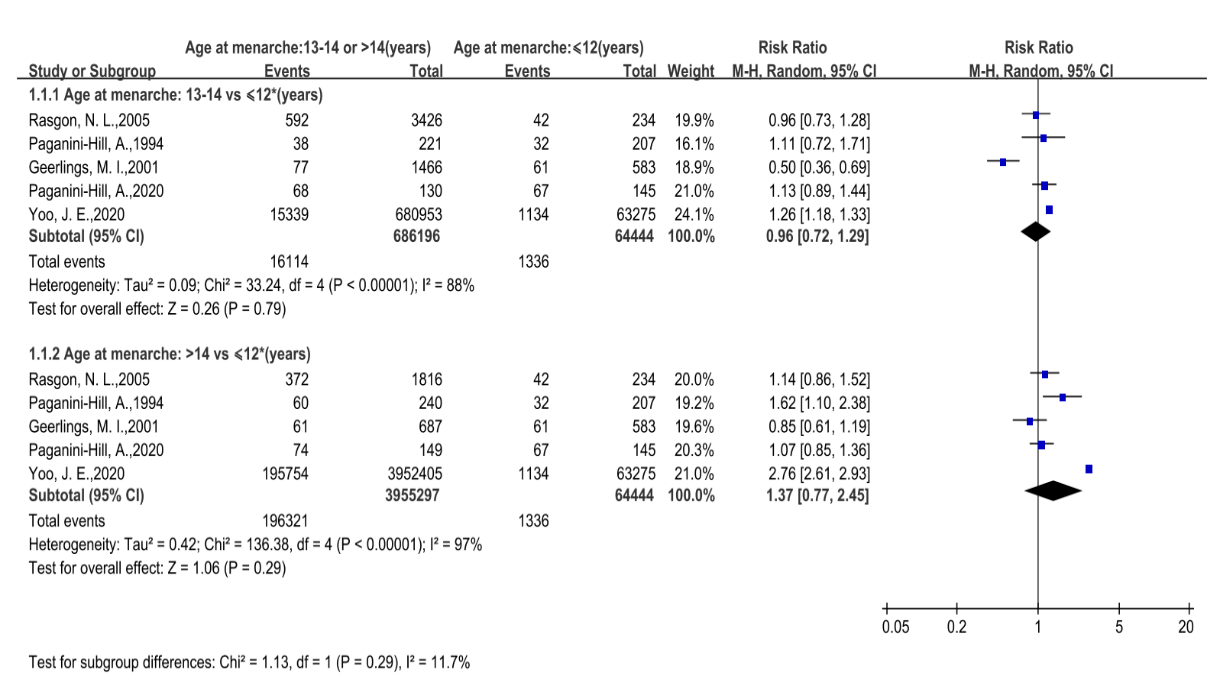


B) AD


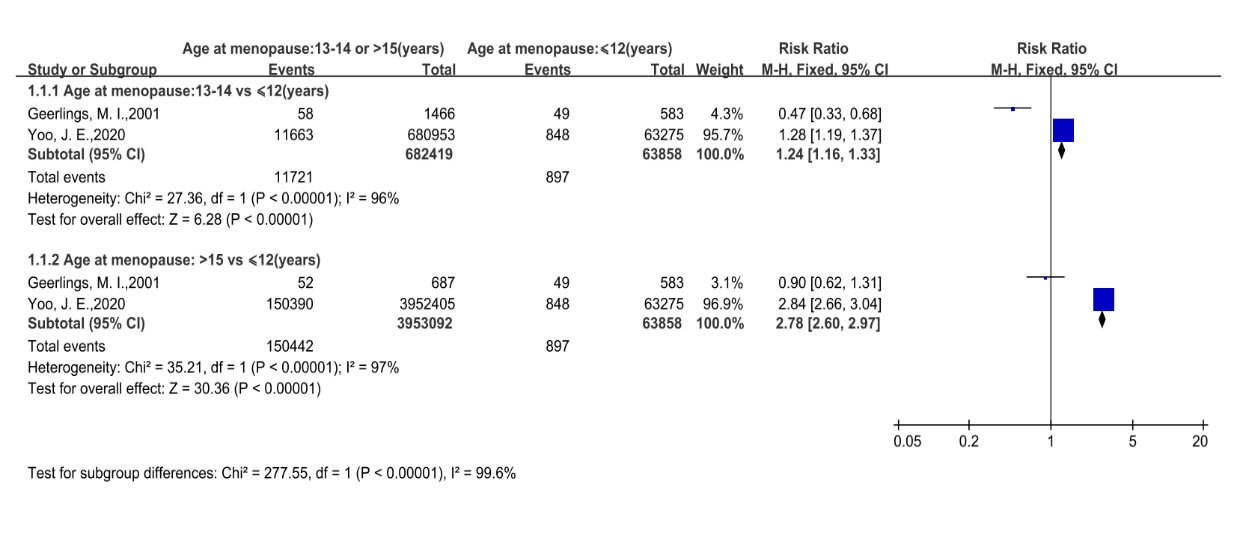


C) CI


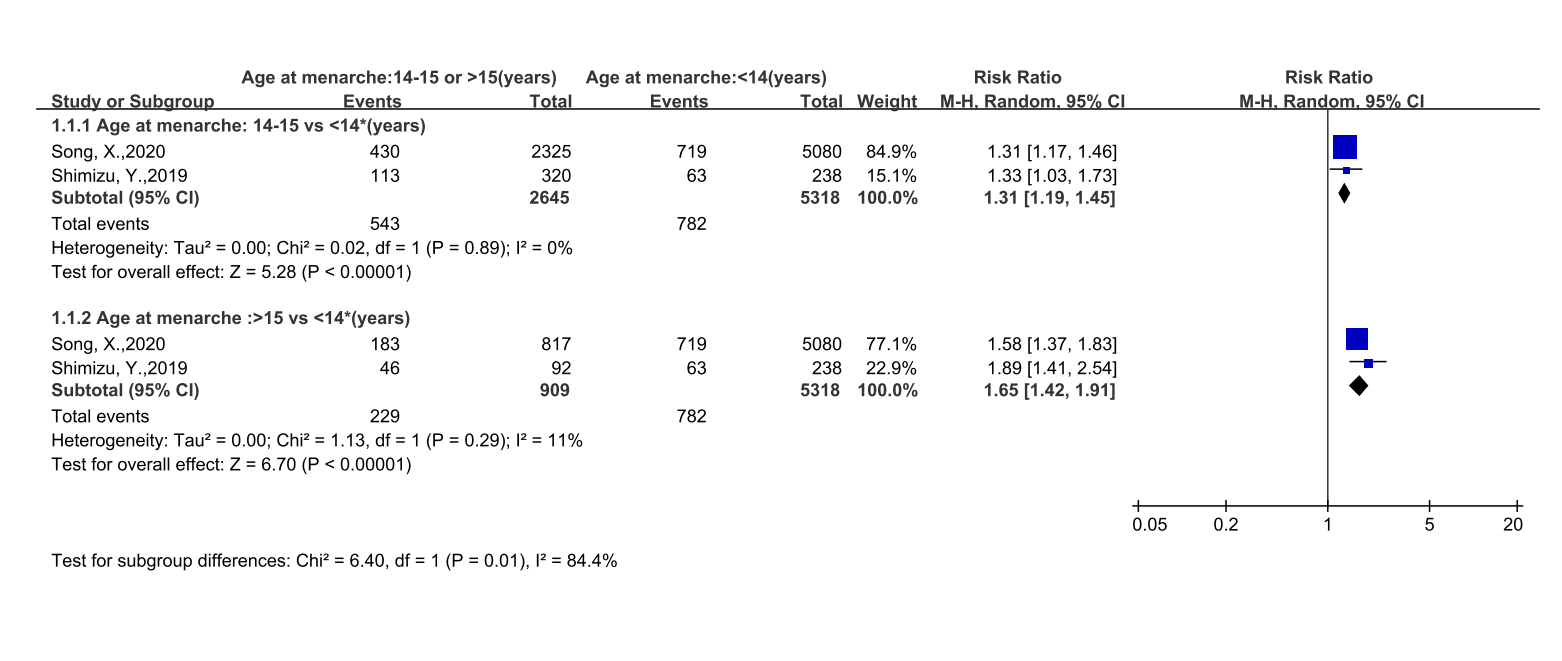


**(2) Age at menopause (≥45 vs＜45* years) and all-cause dementia, AD and CI**

A) All-cause dementia


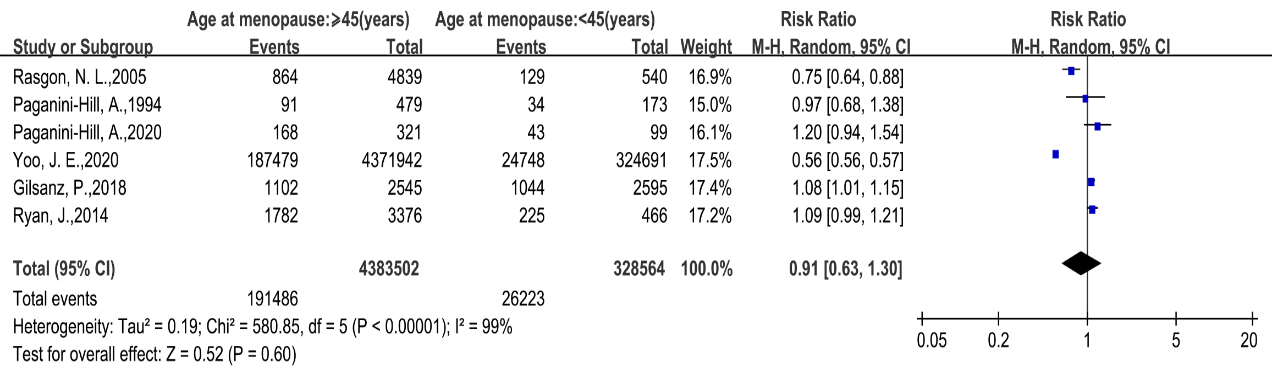

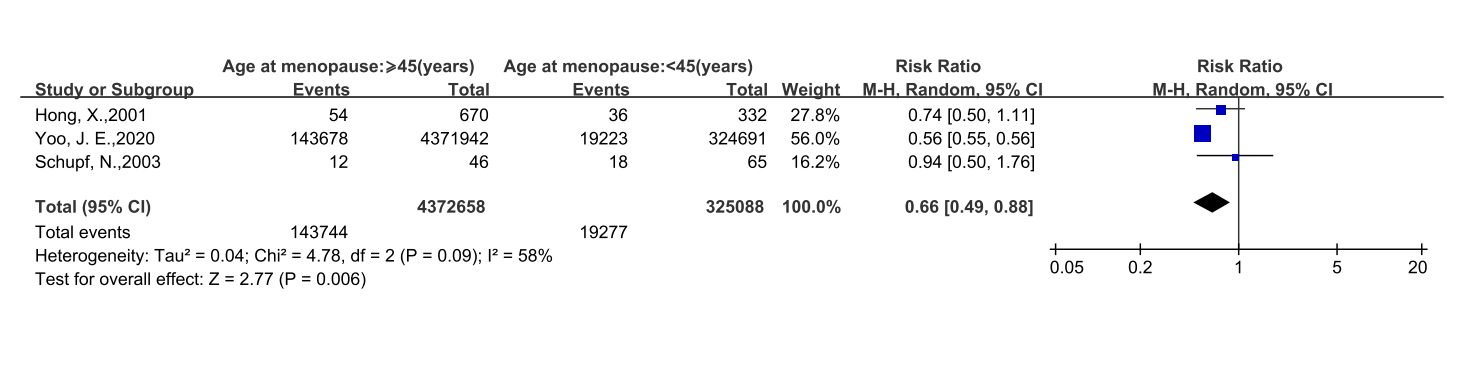


B) AD

C) CI


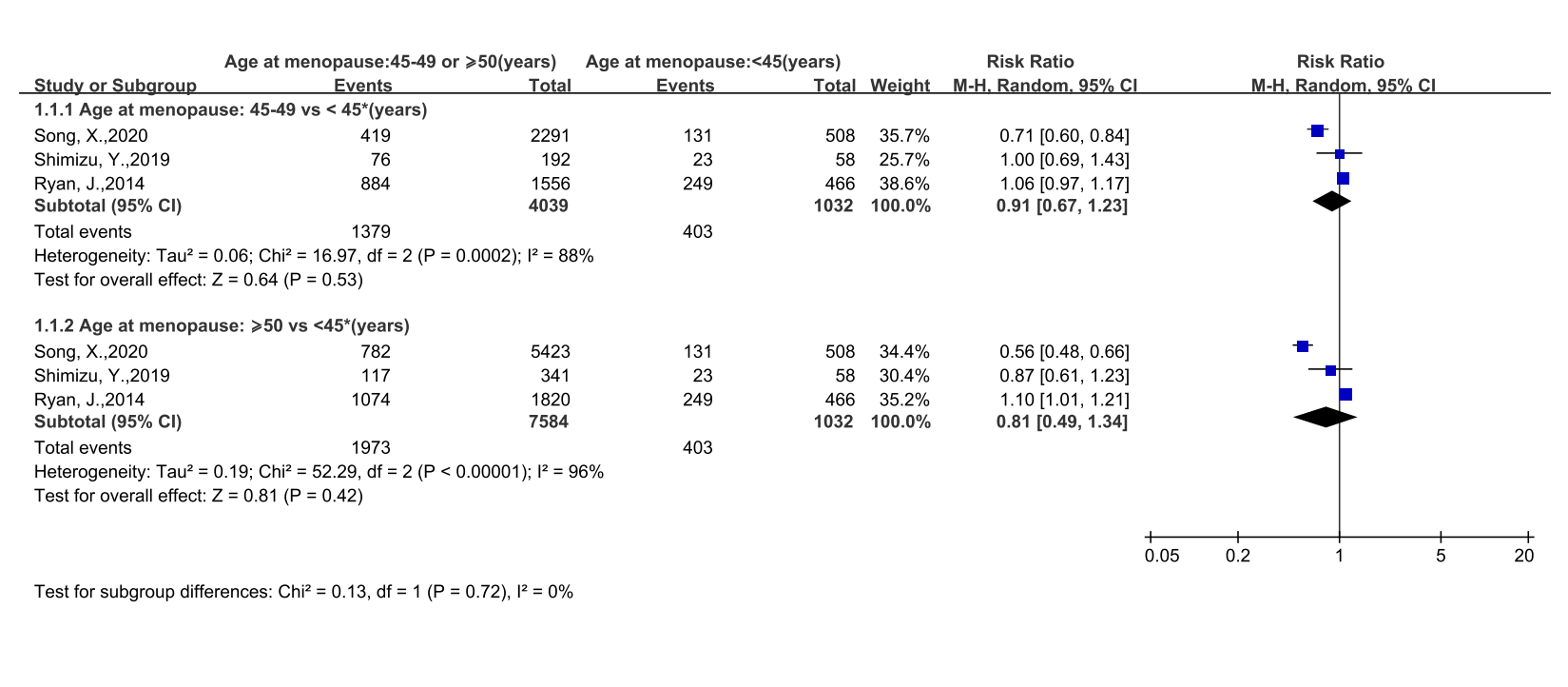


**(3) Reproductive period (≥35 vs <35* years) and all-cause dementia, AD and CI**


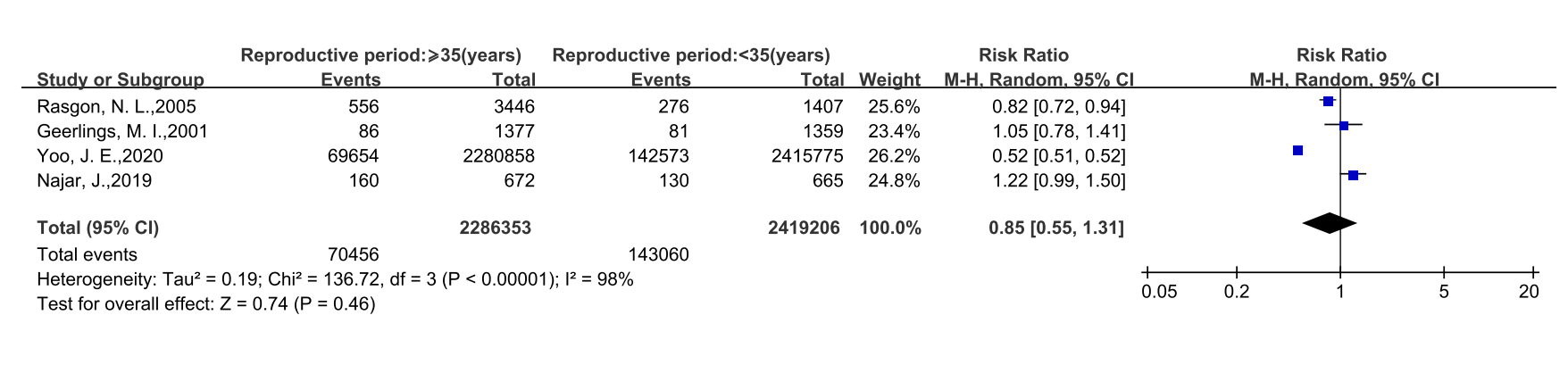


A) All-cause dementia


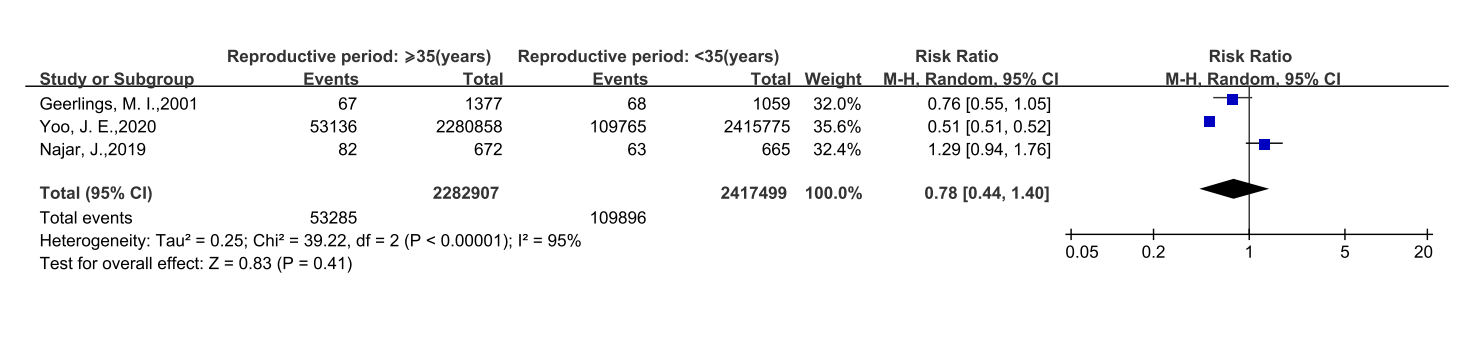


B) AD


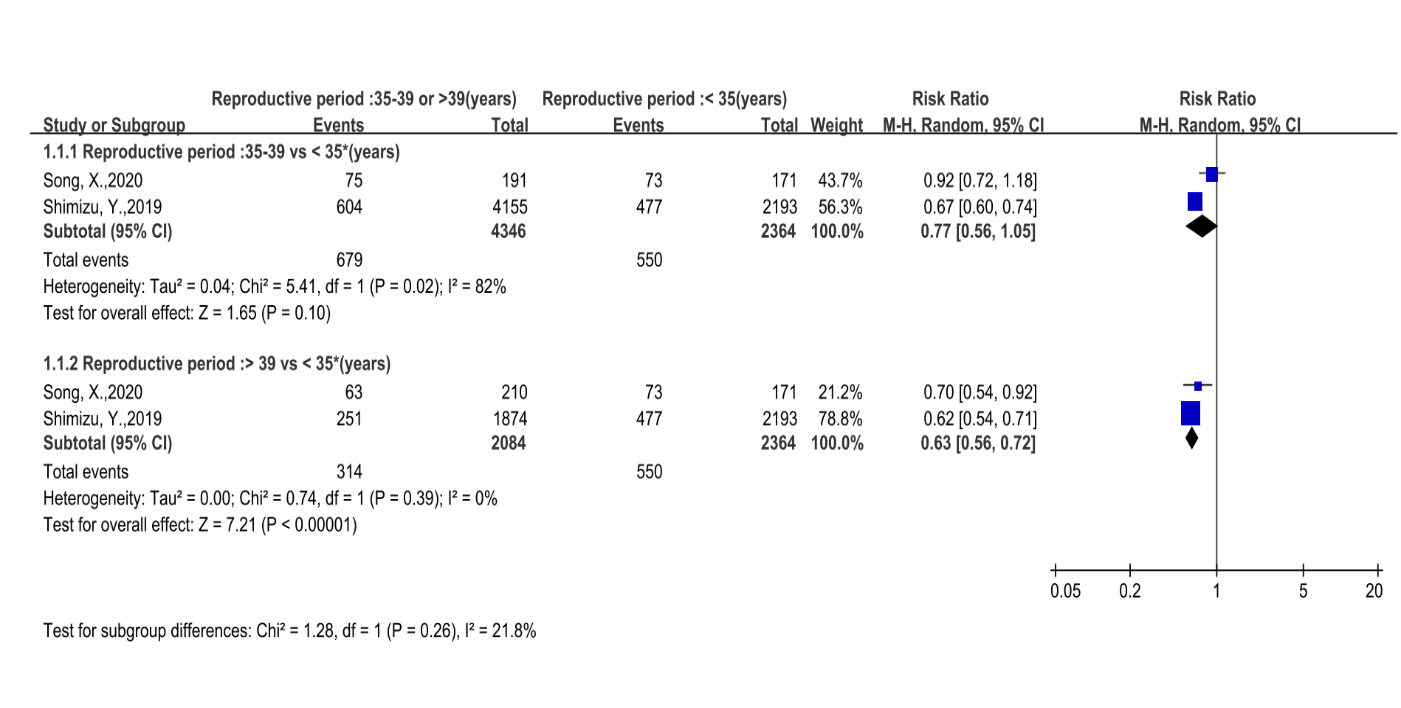


C) CI

**e Figure 7. Funnel Plot**

A) Age at menarche and all-cause dementia


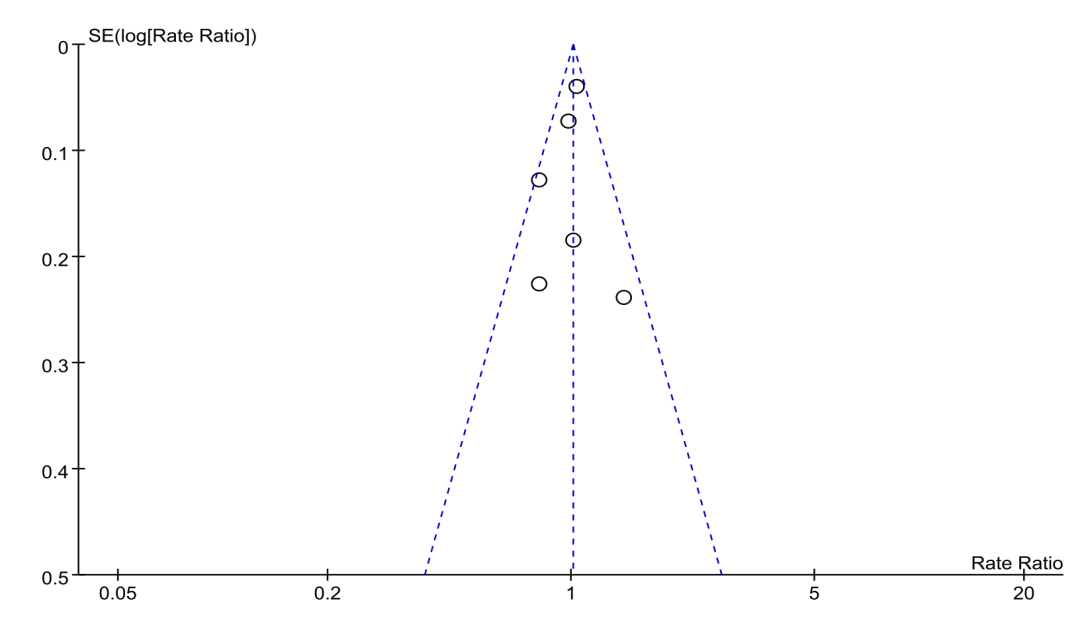


Center dotted line represents the summary effect estimate. Outer dotted lines display 95% CIs. Egger’s test indicated no publication bias (Egger: P=0.851).

B) Age at menopause and all-cause dementia


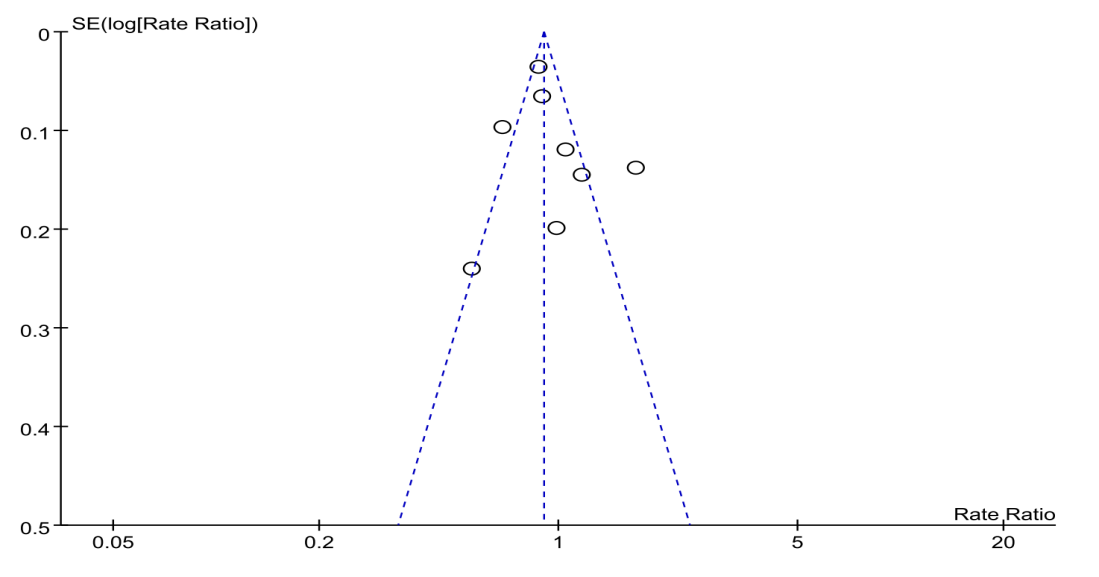


Center dotted line represents the summary effect estimate. Outer dotted lines display 95% CIs. Egger’s test indicated no publication bias (Egger: P=0.711).
